# Trained immunity in respiratory diseases: Mechanisms of action and intervention strategies

**DOI:** 10.1016/j.pccm.2026.02.003

**Published:** 2026-03-16

**Authors:** Szu-yu Lee, Jing Li, Xikun Zhou

**Affiliations:** a State Key Laboratory of Oral Diseases, National Clinical Research Center for Oral Diseases, Chinese Academy of Medical Sciences Research Unit of Oral Carcinogenesis and Management, West China Hospital of Stomatology, Sichuan University, Chengdu, Sichuan 610041, China; b Department of Biotherapy, Cancer Center and State Key Laboratory of Biotherapy, West China Hospital, Sichuan University, Chengdu, Sichuan 610041, China; c Laboratory of Pathogen Research, West China Hospital, Sichuan University, Chengdu, Sichuan 610041, China; d Frontiers Medical Center, Tianfu Jincheng Laboratory, Chengdu, Sichuan 610095, China

**Keywords:** Trained immunity, Innate immune memory, Inflammation modulation, Infectious respiratory diseases, Asthma, Chronic obstructive pulmonary disease, Pulmonary fibrotic diseases, Lung cancer, Bronchiectasis

## Abstract

Trained immunity refers to a form of nonspecific immunological memory established through epigenetic modifications and metabolic reprogramming in innate immune cells following stimulation. This concept offers a novel framework for understanding and treating respiratory diseases. Chronic inflammation and dysregulated immune memory resulting from respiratory immune imbalance underlie many respiratory conditions, including infectious pneumonia, asthma, and chronic obstructive pulmonary disease (COPD). The core mechanisms of trained immunity involve epigenetic regulation—mediated by histone modifications such as histone H3 lysine 4 trimethylation (H3K4me3)—and metabolic reprogramming, exemplified by glycolysis. Trained immunity exhibits a “double-edged sword” effect in respiratory diseases: appropriate activation enhances pathogen clearance, whereas excessive activation may lead to sustained inflammation and tissue damage. Intervention strategies targeting trained immunity—such as vaccine-induced training, metabolic modulation, and natural product application—have shown clinical promise. However, the field faces challenges, including a lack of specific regulatory approaches and clinically applicable biomarkers. Future efforts should focus on deepening mechanistic insights and facilitating the clinical translation of precise interventions, thereby opening new paradigms for the prevention and treatment of respiratory diseases.

## Introduction

Trained immunity is defined as a biological process in which innate immune cells undergo durable functional alterations via epigenetic modifications and metabolic reprogramming following an initial stimulus, resulting in an amplified nonspecific immune response upon subsequent stimulation. The concept originated from early observations of cross-protective effects induced by traditional vaccines. A representative example is Bacillus Calmette-Guérin (BCG) vaccination. In 2003, Garly et al^1^ reported that BCG vaccination in West African children not only conferred protection against tuberculosis but also enhanced resistance to unrelated pathogens, such as tetanus and diphtheria. This heterologous protection could not be explained by classical adaptive immunity, suggesting that the innate immune system may possess a form of “memory-like” function. Subsequent studies explored the underlying molecular mechanisms, culminating in a landmark study by Netea’s team in 2011, which formally introduced the concept of “trained immunity” and established a theoretical foundation for this research field.^2^

The introduction of trained immunity represents a paradigm shift in immunology, challenging the traditional view that only adaptive immunity possesses immunological memory. The hallmark of trained immunity is its capacity for nonspecific memory.^3^ Unlike the highly antigen-specific memory of adaptive immunity, trained immunity confers protection against reinfection independently of T and B lymphocytes.^4^ Moreover, this memory is heterologous—it is not directed against a single pathogen. Instead, it arises from epigenetic and metabolic reprogramming of innate immune cells, which globally enhances cellular responsiveness and alertness. This results in a durable, broad-spectrum defense system capable of providing cross-protection against diverse pathogens.

As the primary interface between the human body and the external environment, the respiratory system has evolved a sophisticated immune defense network. This network comprises physical barriers (e.g., mucociliary clearance, MCC), chemical defenses (e.g., antimicrobial proteins), and various immune mechanisms involving both immune and non-immune cells.^5^^,^^6^ These components collectively enhance respiratory defense upon environmental exposure, reducing infection risk. Despite this complexity, the respiratory tract remains highly susceptible to pathogen invasion due to its direct environmental exposure, imposing a substantial clinical burden. Dysregulation of respiratory immune responses can precipitate a range of high-morbidity and high-mortality diseases, including chronic obstructive pulmonary disease (COPD) and various forms of pneumonia (e.g., community-acquired pneumonia, hospital-acquired pneumonia, and coronavirus disease 2019 [COVID-19]). This creates a significant unmet clinical need. Traditional immunotherapies, such as vaccines and monoclonal antibodies, have notable limitations in treating respiratory diseases, including low response rates and substantial side effects. These challenges are partly attributable to the high heterogeneity of conditions like severe asthma and the complexity of overlapping patient immune profiles.^7^ Currently, research in this field remains in the early stages of translating fundamental mechanistic insights into clinical applications. A disconnect persists among “mechanism, disease, and intervention”, largely due to an insufficient understanding of respiratory disease heterogeneity and inefficient clinical translation.

Given the substantial unmet clinical needs in respiratory medicine and the limitations of conventional therapeutic strategies, researchers are increasingly motivated to move beyond traditional immunological paradigms and explore novel pathological mechanisms and intervention approaches. In recent years, the concept of trained immunity has offered a fresh perspective on dysregulated immune memory in respiratory diseases. Notably, epidemiological observations during the COVID-19 pandemic revealed significantly lower mortality rates in regions with routine BCG vaccination compared to non-vaccinated areas,^8^ sparking intense interest in the heterologous protective effects of BCG.^9^ This review aims to systematically delineate the role and translational potential of trained immunity in respiratory diseases. We first dissect the core mechanisms underlying trained immunity, followed by an in-depth discussion of its involvement in the pathogenesis and progression of specific respiratory conditions, including COPD, asthma, pulmonary infections, and lung cancer. Building on this mechanistic foundation, we outline and evaluate potential intervention strategies that target trained immunity, with the goal of identifying novel therapeutic avenues and reducing the burden of trained immunity-associated respiratory diseases. Finally, we address current challenges in the field—such as the specificity of regulation, heterogeneity in the training of distinct pulmonary immune cell populations, and hurdles in clinical translation—and offer perspectives on future research directions and clinical applications.

## Molecular and cellular basis of trained immunity

The molecular and cellular underpinnings of trained immunity involve functional remodeling of immune cells and the coordinated action of multidimensional regulatory mechanisms, including epigenetic reprogramming, metabolic rewiring, and signaling pathway activation. Core immune populations serve as primary effectors in the induction and maintenance of trained immunity; their phenotypic and functional alterations initiate downstream regulatory cascades. In this section, we first examine these core immune cells and their central roles in establishing and executing trained immunity.

### Core immune cells

The canonical effector cells of trained immunity include monocytes, macrophages, and natural killer (NK) cells. Subsequent studies have extended this concept to other cell types, including microglia, hematopoietic stem cells, and epithelial cells, all of which exhibit features of innate immune memory.^10^ Inducers of trained immunity comprise pathogen-associated molecular patterns (PAMPs), damage-associated molecular patterns (DAMPs), and specific metabolites. These ligands are recognized by pattern recognition receptors (PRRs) on innate immune cells, triggering intracellular signaling cascades, cellular reprogramming, and ultimately, the establishment of immunological memory.

During β-glucan (a major cell wall component of *Candida albicans*)-induced trained immunity in monocytes and macrophages, epigenetic reprogramming occurs, characterized by increased histone H3 lysine 4 trimethylation (H3K4me3) and histone H3 lysine 27 acetylation (H3K27ac) enrichment.^11^ These histone modifications promote transcription of antibacterial response genes and leave specific chromatin signatures. Although some marks are erased upon stimulus withdrawal, residual histone modifications facilitate rapid and enhanced transcription of marked genes upon secondary stimulation, resulting in an amplified immune response.^12^ Myeloid-derived suppressor cells (MDSCs) and neutrophils—typically associated with immunosuppression or acute inflammation—also undergo functional remodeling during training. MDSCs may modulate their T-cell suppressive capacity,^13^ while neutrophils can acquire enhanced neutrophil extracellular trap (NET) formation or extended lifespan.^14^ Both cell types contribute to regulating the intensity and duration of inflammatory responses. Additionally, crosstalk between innate lymphoid cells (ILCs) and airway epithelial cells represents another key axis of trained immunity. Upon sensing alarm signals, activated ILCs secrete interleukin (IL)-13 (IL-13),^15^ inducing a “trained-like” state in epithelial cells. This state persistently elevates baseline epithelial defense, enhances barrier function, and enables more effective coordination of innate and adaptive immune responses upon subsequent challenges. Collectively, these cellular events establish a strengthened, “trained” immune frontline at mucosal surfaces.

Different cell types exhibit marked specificity and variability in the induction and maintenance of trained immunity. During the induction phase of macrophage training, glycolytic metabolism shifts from aerobic oxidation (in the resting state) to glycolysis, with oxidative phosphorylation suppressed.^16^ This shift is accompanied by epigenetic reprogramming, such as enrichment of activating histone marks H3K4me3 and H3K27ac at the promoters of key glycolytic enzymes.^17^ Upon secondary stimulation, trained cells display a renewed glycolytic burst, significantly increased secretion of pro-inflammatory cytokines (e.g., tumor necrosis factor-α [TNF-α], IL-6, IL-1β), and enhanced phagocytic activity.^18^

The pattern of trained immunity in neutrophils differs from the relatively slow, persistent, and epigenetically dependent mode observed in alveolar macrophages. The induction phase in neutrophils is more rapid but transient. A study by Criado et al^19^ found that immunization with inactivated or attenuated oral *Mycobacterium avium* subspecies *paratuberculosis* (Map) vaccines enhanced phagocytosis of Map and induced increased NETosis against Map in rabbit neutrophils. However, immunization with an inactivated vaccine (Silirum^Ⓡ^) did not induce changes in NETosis, phagocytosis, or other functions in goat neutrophils. This indicates that not all stimuli can induce trained immunity in neutrophils; such induction is stimulus-specific, varies across species, and is often difficult to detect, clearly demonstrating the cell-type specificity of trained immunity.

**Fig. 1:**
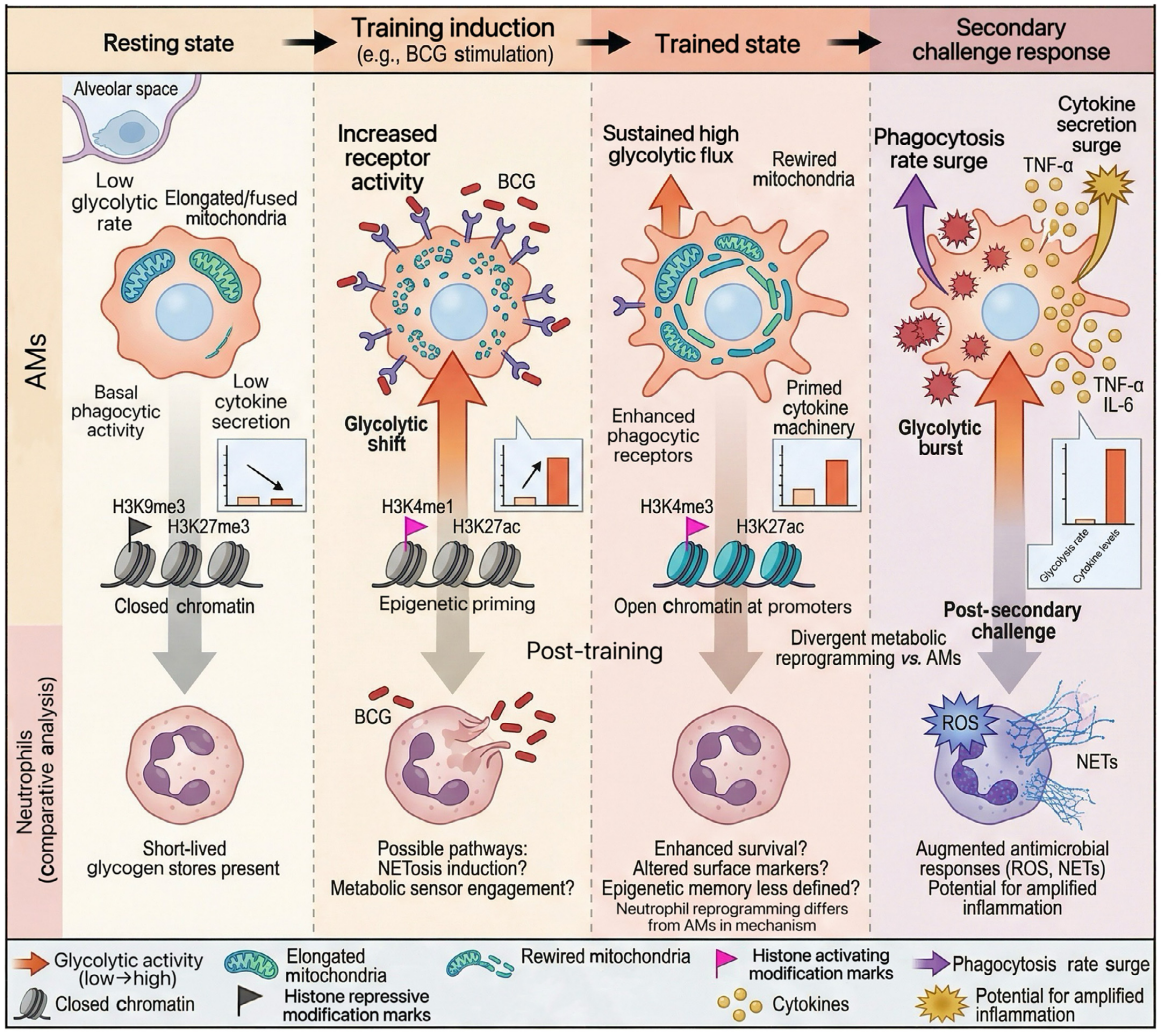
The training process and phenotypic changes of lung immune cells. Temporal dynamics of trained immunity in pulmonary immune cells (metabolic, functional, and epigenetic remodeling): In the resting state, alveolar macrophages exhibit low glycolytic rates, elongated/fused mitochondria, and H3K9me3/H3K27me3-modified closed chromatin, with basal phagocytic and cytokine secretion activities; neutrophils have sufficient glycogen stores but short lifespans. Upon BCG-induced training, alveolar macrophages show increased receptor activity, a glycolytic shift, and H3K4me1/H3K27ac-mediated epigenetic priming; neutrophils may participate via metabolic sensing or epigenetic induction. In the trained state, alveolar macrophages sustain high glycolytic flux, mitochondrial rewiring (e.g., elongation), and H3K4me3/H3K27ac-modified open chromatin at promoters, with activated phagocytic receptors and cytokine machinery; neutrophils display enhanced survival, altered surface features, and divergent metabolic reprogramming from alveolar macrophages. Upon secondary challenge, alveolar macrophages undergo phagocytosis surges and glycolytic bursts, secreting TNF-α and IL-6; neutrophils release reactive oxygen species and extracellular traps to enhance antimicrobial responses, with potential for amplified inflammation. AMs, Alveolar macrophages; BCG, Bacillus Calmette–Guérin; H3K9me3, Histone 3 lysine 9 trimethylation; H3K27ac, Histone 3 lysine 27 acetylation; H3K27me3, Histone 3 lysine 27 trimethylation; H3K4me1, Histone 3 lysine 4 monomethylation; H3K4me3, Histone 3 lysine 4 trimethylation; IL-6, Interleukin-6; NETs, Neutrophil extracellular traps; NETosis, Neutrophil extracellular trap-mediated cell death; ROS, Reactive oxygen species; TNF-α, Tumor necrosis factor-alpha.

In summary, trained immunity exhibits pronounced cell-type specificity. Monocytes and macrophages primarily rely on coupled metabolic and epigenetic reprogramming to establish long-term inflammatory memory. In contrast, neutrophils—despite their short lifespan—undergo more transient but functionally significant reprogramming (Fig. 1). These mechanistic distinctions have direct therapeutic implications, indicating that cell-targeted approaches are necessary for effective and safe modulation of trained immunity in respiratory diseases.

### Epigenetic reprogramming mechanisms

Epigenetic reprogramming is defined as persistent changes in transcriptional programs and cellular physiology that do not involve permanent genetic alterations such as mutations or recombination.^4^ It refers to the regulation of transcription, DNA replication, and other genomic functions without altering the DNA sequence, playing a crucial role in energy metabolism and gene expression regulation.^20^ Epigenetic reprogramming serves as the molecular foundation of trained immunity, encompassing histone modifications, chromatin accessibility, and metabolic signaling, which collectively determine the expression profiles of immune genes. This makes the entire process far more complex than a single modification event.

Innate immune cells maintain high expression levels of positive signaling transducers and regulators to effectively respond to pathogens, while the transcription of negative regulatory factors is also modulated to preserve innate immune homeostasis and resolve inflammatory responses. The expression of many such genes is controlled by histone modifications. At the histone modification level, common epigenetic marks involve histone lysine residues—particularly methylation-related marks such as H3K4me3 and H3K4me1, and acetylation-related marks such as H3K27ac. These are associated with the expression of pro-inflammatory cytokine genes, including tumor necrosis factor-alpha (*TNFA*) and IL-6 (*IL6*).^21^ Such modifications can alter chromatin spatial conformation, maintaining chromatin in an open state and reducing spatial resistance to transcription initiation, thereby facilitating rapid and efficient transcription of target genes. This ultimately endows cells with immunological memory. Inhibitory histone methylation marks also play key regulatory roles in trained immunity. A study in a *Drosophila* model confirmed that after training with heat-killed Gram-negative bacteria, H3K9me3 levels at the promoter of the *PGRP-SC* gene in the fly gut were significantly elevated, leading to downregulated expression of this negative regulator.^22^ This relieved suppression on downstream antimicrobial peptide genes, enhancing the fly’s bactericidal capacity upon reinfection. Novel modifications such as histone lactylation have also been recently discovered.^23^ After BCG vaccination, lactate produced from metabolic reprogramming drives lactylation at the H3K18 site. This modification, catalyzed by E1A-binding protein p300/CREB-binding protein (p300/CBP), persists long-term in enhancer regions, promoting the transcription of inflammatory genes such as *Il1b*. This epigenetic reprogramming enhances the rapid recall response of innate immune cells upon secondary challenge, thereby strengthening early pathogen clearance and contributing to host protection.

Histone modifications not only locally affect chromatin accessibility but can also trigger systematic remodeling of three-dimensional chromatin structure across the entire genome. A core mechanism of this remodeling involves the anchoring and organization of chromatin in nuclear space by nuclear lamins. Studies show that deficiency or dysfunction of lamin A/C disrupts nuclear envelope integrity, causing dissociation of heterochromatic regions (lamin-associated domains, LADs) that were originally tightly anchored at the nuclear periphery.^24^ DNA accessibility in these released chromatin regions significantly increases, accompanied by loss of inhibitory histone marks like H3K9me3 and gain of activating marks. These changes in the nuclear environment provide far greater access for reprogramming factors and transcriptional machinery than before, greatly accelerating the cellular epigenetic reprogramming process.

Many epigenetic enzymes utilize energy metabolites as essential cofactors. Acetyl coenzyme A (Acetyl-CoA) serves as the specific substrate for histone acetyltransferases, providing the acetyl groups required for acetylation marks such as H3K27ac.^10^ S-adenosylmethionine (SAM) functions as a methyl donor, supplying methyl groups for histone methyltransferase-mediated methylation marks such as H3K4me3, thereby regulating the deposition efficiency of methylation marks on chromatin.^20^^,^^25^ The close link between epigenetic regulation and metabolic processes enables immune cells to integrate endogenous metabolic signals, leading to long-term, stable remodeling of cellular transcriptional programs and ultimately completing immune training.

The dynamic regulation of DNA methylation is also a key layer of epigenetic reprogramming in trained immunity. Glutaminolysis leads to the accumulation of succinate and α-ketoglutarate. α-Ketoglutarate is a cofactor for important epigenetic enzyme families involved in DNA demethylation reactions.^26^ This process does not directly remove methyl groups; instead, it dynamically and reversibly regulates methylation status by progressively oxidizing 5-methylcytosine (5mC) to intermediates such as 5-hydroxymethylcytosine (5hmC).^27^ This significantly enhances chromatin accessibility, laying the groundwork for transcription factor binding and subsequent gene transcription. In addition to its dynamic regulation, DNA methylation has emerged as a critical determinant of the long-term stability and qualitative outcome of trained innate immune memory. Recent evidence indicates that persistent DNA methylation remodeling at promoters and enhancers of inflammatory and metabolic genes underlies the formation of distinct trained immunity states, including persistent innate exhaustion memory and low-grade inflammatory memory.^28^ These memory states are characterized, respectively, by attenuated responsiveness following chronic stimulation and by sustained, subclinical inflammatory activity. Compared with histone modifications, DNA methylation provides a more stable epigenetic layer, allowing trained innate immune cells to maintain long-lasting transcriptional programs even after withdrawal of the initial stimulus. This stability may explain how trained immunity shifts from an initially protective response to maladaptive immune exhaustion or chronic inflammation under prolonged environmental or metabolic stress.^28^

Beyond the DNA and histone levels, epigenetic reprogramming in trained immunity also involves the dynamic regulation of RNA structure. Recent research developed an ultra-low-input RNA structure probing method,^29^ revealing a novel regulatory mode for Regnase-1 in macrophages. Regnase-1, an RNase, does not randomly degrade the mRNAs of pro-inflammatory factor-encoding genes. Instead, it precisely cleaves and regulates the stability of these mRNAs by recognizing the specific secondary structures (e.g., stem-loop structures) of the mRNAs encoded by genes for pro-inflammatory factors such as IL-6 and TNF-α. While traditional histone modifications ensure transcriptional activation of pro-inflammatory genes, Regnase-1-mediated RNA structure regulation determines the lifespan of the transcripts. Together, they maintain an appropriate and persistent pro-inflammatory state in trained immunity, avoiding tissue damage from overactivation. Moreover, the ultra-low-input nature of this technique enables the study of RNA structural dynamics in rare pulmonary immune cells (e.g., alveolar macrophages, trained dendritic cells), overcoming the limitation of traditional methods requiring large cell samples.

### Metabolic reprogramming mechanisms

The metabolic reprogramming mechanism of trained immunity is the cytological basis for establishing immunological memory. Its core lies in profound shifts in cellular energy metabolic pathways. After receiving the primary stimulus, innate immune cells rapidly shift their metabolic mode from efficient oxidative phosphorylation to a predominant reliance on glycolysis. This phenomenon is analogous to the “Warburg effect” in cancer cells.^30^ Even under sufficient oxygen, cells prioritize glycolysis for rapid adenosine triphosphate (ATP) production while providing precursor molecules for biosynthesis. This metabolic switch not only meets energy demands but also supports the massive synthesis of macromolecules (nucleic acids, proteins, lipids) to fuel cell proliferation, cytokine production, and the deposition of epigenetic modifiers. This process is centrally regulated by the protein kinase B (Akt)/mammalian target of rapamycin (mTOR)/hypoxia-inducible factor 1α (HIF-1α) signaling axis: Activated Akt signaling promotes the synthesis of various glycolytic enzymes via the mTOR complex 1 (mTORC1) pathway and can stabilize the key transcription factor HIF-1α. HIF-1α upregulates the expression of numerous glycolytic genes, thereby consolidating the glycolytic metabolic state.^31^

Beyond shifts in energy metabolism pathways, metabolic intermediates also participate as key signaling molecules in trained immunity regulation. Cholesterol synthesis pathway intermediates like mevalonate or branch pathway products such as 24(S),25-epoxycholesterol (24(S),25-EC) are also actively involved.^32^ Some intermediates of the mevalonate pathway, like farnesyl pyrophosphate (FPP), can themselves act as signaling molecules.^33^ For example, FPP serves as a donor for protein post-translational modifications. Other key metabolites also play important regulatory roles in trained immunity. For instance, the tricarboxylic acid (TCA) cycle intermediate aconitate can inhibit inflammatory responses by activating the transcription factor nuclear factor erythroid 2-related factor 2 (Nrf2),^34^ while succinate accumulation stabilizes HIF-1α,^35^ forming a positive feedback loop regulating glycolysis.

This multi-layered metabolic remodeling has direct links with epigenetic reprogramming. Specific metabolites directly drive changes in epigenetic modifications, providing the molecular basis for enhanced cellular function in trained immunity. Acetyl-CoA, produced from glycolysis and the TCA cycle, provides the acetyl groups required for histone acetylation, thereby promoting an open chromatin configuration at inflammatory gene loci. In parallel, increased availability of SAM from one-carbon metabolism supports histone and DNA methylation processes,^36^ including activating marks such as H3K4me3 that are characteristic of trained immunity. Meanwhile, α-ketoglutarate derived from the TCA cycle functions as a critical cofactor for histone and DNA demethylases, facilitating the dynamic removal of repressive methylation marks. Together, these metabolite-driven modifications reshape the chromatin landscape, establishing a permissive epigenetic state that enhances transcriptional responsiveness upon secondary stimulation and underlies the functional phenotype of trained immunity.

Metabolic reprogramming also exhibits significant spatiotemporal heterogeneity. The focus of metabolic reprogramming differs among cell types such as macrophages, dendritic cells, and NK cells. Temporally, metabolic changes immediately after the primary stimulus are intense and dramatic. During the establishment and maintenance phases of trained immunity, cells may shift to relying on oxidative phosphorylation to sustain long-term functional potential. Studies indicate that enhancing pentose phosphate pathway (PPP) activity by intervening with pyruvate kinase M2 (PKM2) (a key glycolytic enzyme) can drive CD8+ T cells toward a more memory-like, tumor-resistant T cell factor 1 (TCF1)+ progenitor phenotype,^37^ suggesting that appropriately reducing glycolysis may favor the formation of long-term immunological memory. Ultimately, this dynamic, multi-layered metabolic remodeling provides the necessary energy, substrates, and signals for epigenetic reprogramming and enhanced cellular function in trained immunity, enabling cells to mount a more robust response upon secondary stimulation.

Beyond their mechanistic role in trained immunity establishment, metabolic pathways such as glycolysis and the mTOR/5′-adenosine monophosphate-activated protein kinase (AMPK) axis represent actionable therapeutic targets in respiratory diseases. Enhanced glycolytic flux supports rapid cytokine production and epigenetic remodeling in trained innate immune cells; however, sustained glycolysis has been associated with chronic inflammatory phenotypes in asthma and COPD. Targeting key glycolytic enzymes or upstream regulators may therefore attenuate pathological immune training while preserving host defense. mTOR signaling promotes anabolic metabolism and inflammatory activation, whereas AMPK functions as a metabolic checkpoint limiting excessive immune activation. Pharmacological modulation of the mTOR/AMPK balance has demonstrated the capacity to fine-tune trained immunity, offering a strategy to either enhance antimicrobial responses or suppress maladaptive inflammation.^31^^,^^38^^,^^39^ Collectively, these findings suggest that selective targeting of metabolic pathways may enable precise regulation of trained immunity intensity, providing a rational framework for therapeutic intervention in respiratory diseases.

### Signaling pathway activation

The establishment of trained immunity begins with the precise recognition of pathogen-associated molecules by pattern recognition receptors. This process involves cell membrane Toll-like receptors (TLRs) and cytosolic nucleotide-binding oligomerization domain (NOD)-like receptors. NOD1 and NOD2 recruit the adaptor protein receptor-interacting serine/threonine-protein kinase 2 (RIP2) via caspase recruitment domain (CARD)–CARD domain interactions, forming a complex that further recruits various tumor necrosis factor receptor-associated factor (TRAF) family proteins including TRAF2, TRAF5, and TRAF6, thereby activating nuclear factor-κB (NF-κB) and mitogen-activated protein kinase (MAPK) signaling pathways.^40^ This drives the expression of multiple pro-inflammatory cytokines and chemokines. Research shows that *Streptococcus pneumoniae* (*S. pneumoniae*) induces NF-κB activation via the NOD2 receptor, participating in immune defense responses against *S. pneumoniae* infection in lung tissue.^41^

The activation of NF-κB and MAPK in trained immunity differs from conventional transient immune responses in that these signaling pathways are coupled with persistent metabolic and epigenetic reprogramming. Initial stimuli (e.g., BCG, β-glucan) drive a metabolic switch to glycolysis by activating the mTOR–HIF-1α axis. Accumulated metabolites such as acetyl-CoA can directly serve as substrate donors, catalyzing and maintaining H3K27ac modifications at the promoter regions of NF-κB target genes (e.g., those encoding inflammatory cytokines), keeping chromatin in an open state.^31^ mTOR signaling can also phosphorylate and regulate other epigenetic factors, collectively maintaining the sustained accessibility of genes associated with the NF-κB pathway, ensuring the long-term persistence of the trained immune response.

Myeloid differentiation primary response 88 (MyD88) adapter-like protein (Mal) participates in toll-like receptor (TLR) 2 and TLR4 signal transduction, connecting MyD88 to the receptor complex. Upon TLR2 and TLR4 stimulation, Mal and TRAF6 directly interact, which is crucial for TLR2- and TLR4-mediated NF-κB pro-inflammatory responses.^42^ Simultaneously, TLR signaling can activate MAPK family members downstream of NF-κB, such as p38, c-jun N-terminal kinase (JNK), and extracellular signal-regulated kinase 1/2 (ERK1/2),^43^ collectively promoting the production of pro-inflammatory mediators. The synergistic activation of TLR signaling and cytokine networks can also directly regulate macrophage functional polarization. Research demonstrates that leucine-rich repeat kinase 2 (Lrrk2) regulates macrophage function by promoting M1 polarization.^44^ Upon lipopolysaccharide (LPS) activation, Lrrk2 prevents TLR4 from binding to the negative regulator ST2, maintaining sustained NF-κB activation and further consolidating the M1 phenotype. The coordinated activation of these different pathways constitutes the initial signaling foundation for trained immunity, inducing an inflammatory cytokine environment, metabolic alterations, and epigenetic reprogramming, collectively enhancing the specificity and potency of subsequent immune responses.

In addition to MyD88-dependent signaling, emerging evidence suggests that TRIF-related adaptor molecule (TRAM) (encoded by the *TICAM2* gene) may function as an upstream adaptor contributing to the initiation of trained immunity-related programs. Unlike Mal/MyD88, which primarily mediate IL-1-associated inflammatory signaling, TRAM is uniquely characterized by covalent lipid modification that anchors it to cellular membranes, enabling it to integrate membrane stress signals. Through coupling membrane-associated stress sensing with TRIF-dependent pathways, TRAM-associated signaling has been implicated in the initiation of metabolic and epigenetic remodeling processes linked to persistent innate immune responses.^45^^,^^46^

During signal transduction, the balanced regulation of cellular energy status sensors mTOR and AMPK plays a central role. mTOR can reconfigure cellular metabolism, regulating protein translation, cytokine responses, antigen presentation, macrophage polarization, and cell migration. mTORC1 activation is crucial for the activation and function of cells like macrophages; it drives anabolism by upregulating glycolysis, promoting mitochondrial biogenesis and protein synthesis, maintaining the activated state of cells.^39^ In β-glucan-induced trained immunity, mTOR mediates aerobic glycolysis via HIF-1α, forming the metabolic basis of trained immunity.^31^ Mevalonate pathway-induced trained immunity also depends on mTOR activation. In contrast to the anabolic role of mTOR, AMPK is activated during energy deficiency, primarily promoting catabolism to maintain energy supply. During the initiation phase of trained immunity, intracellular AMPK activity is often suppressed.^47^ This inhibition relieves AMPK’s negative regulation of mTORC1, further amplifying mTOR-driven anabolic signals. AMPK can inhibit mTORC1 activity by phosphorylating tuberous sclerosis complex 2 (TSC2) and Raptor; therefore, suppressing AMPK activity is important for strengthening and prolonging the effects of trained immunity.^48^ The activation of mTOR coupled with the inhibition of AMPK constitutes a molecular switch that reshapes cellular metabolism to provide the necessary energy and biosynthetic precursors for epigenetic reprogramming and the establishment of trained immunity.

This process also relies on the synergistic action of cytokine networks, with IL-1β occupying a central position.^49^ IL-1β is both a terminal effector and a key signal amplifier in trained immunity. It can re-activate the NF-κB pathway via its receptor interleukin-1 receptor (IL-1R). NF-κB activation induces microtubule depolymerization, which in turn prevents microtubule-mediated trafficking of stimulator of interferon genes (STING) to the microtubule organizing center and its subsequent degradation, significantly enhancing and prolonging STING-mediated immune responses,^50^ forming a positive feedback loop. Other inflammatory cytokines like TNF-α and interferon-γ (IFN-γ) can also synergize with IL-1β, creating a potent pro-inflammatory environment that collectively enhances the intensity and prolongs the duration of the trained immune response. These cytokines do not merely amplify signals; they also form a mnemonic cycle by regulating metabolism and epigenetics. IL-1β can activate the key glycolytic enzyme PKM2, promoting the accumulation of metabolic intermediates and providing substrates for epigenetic modifications. TNF-α and IFN-γ can induce the expression of histone methyltransferase SET domain-containing protein 7 (SET7/9), enriching H3K4me1 modifications at the promoter regions of NF-κB target genes.^51^ This allows these genes to maintain low-level expression even after the initial stimulus subsides. This cycle is a core feature distinguishing trained immunity from conventional immunity and is key to the persistence of immunological memory.

The establishment and maintenance of trained immunity rely on the coordinated reprogramming of multiple core molecular mechanisms within cells, which can be broadly summarized as three modules: “epigenetic regulation”, “metabolic reprogramming,” and “signaling pathway activation”. In the trained immunity process of alveolar macrophages, LPS (a ligand for TLR4) stimulation alters PKM2 activity in macrophages, shifting from the high-activity tetramer form to the low-activity dimer form. This leads to increased glycolytic flux and promotes the expression of inflammatory cytokines like IL-1β and IL-6.^52^ Accumulated metabolic intermediates (e.g., phosphoenolpyruvate) provide precursors for nucleotide and amino acid synthesis, offering the material and energy basis for rapid cell activation and proliferation. These changes are ultimately consolidated long-term through epigenetic regulation. Metabolic intermediates generated from enhanced glycolysis regulate modifications like histone acetylation and methylation, keeping chromatin at immune-related genes in a persistently open state. Concurrently, transcription factors induced by signaling pathway activation recruit epigenetic regulatory complexes, forming stable epigenetic imprints at target gene promoter regions. This pattern endows conventional signaling pathways the sustained functional changes characteristic of trained immunity, rather than triggering only transient immune activation.

**Fig. 2:**
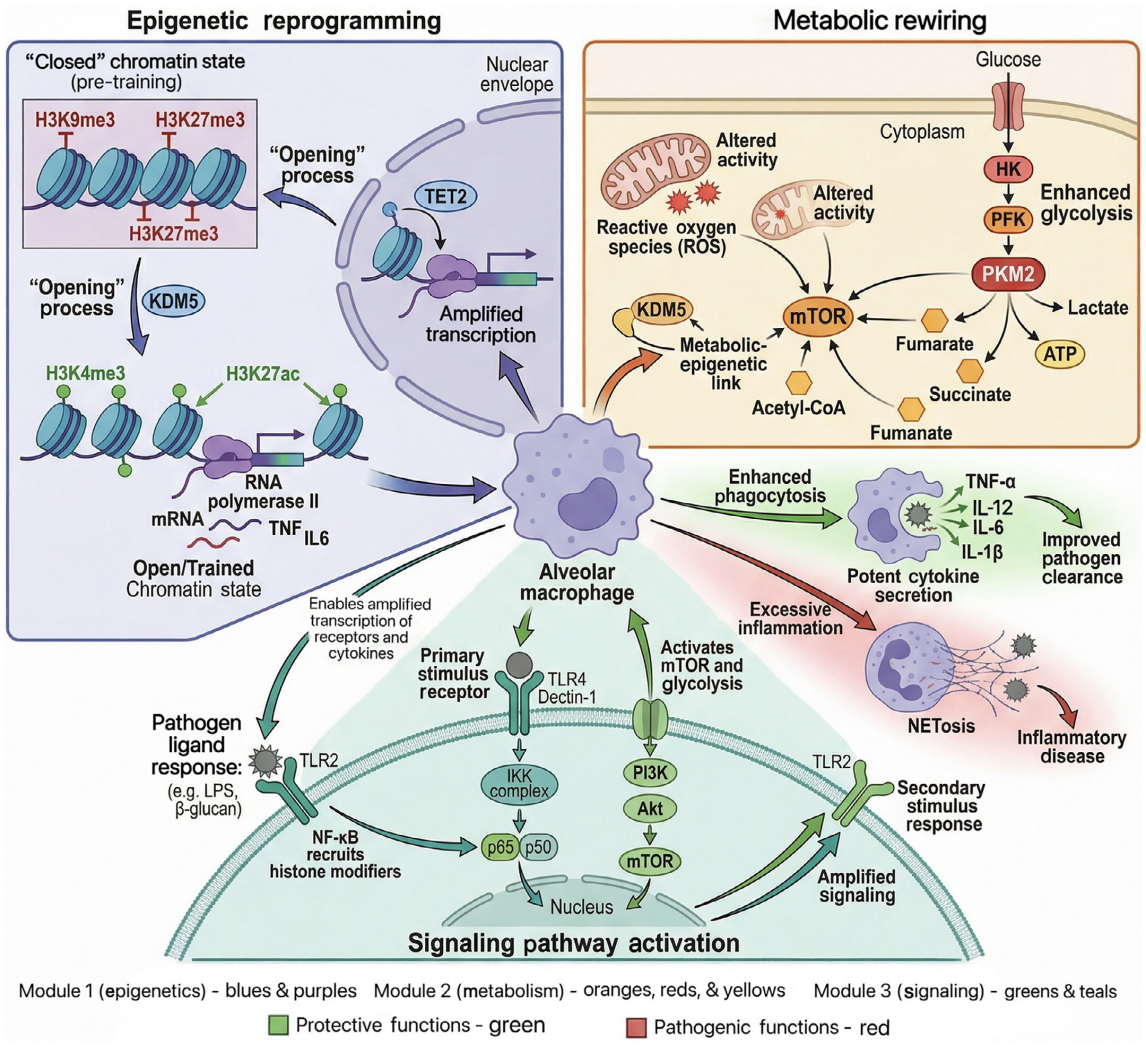
Core molecular mechanisms of trained immunity. Integrated molecular mechanisms of trained immunity in alveolar macrophages: interplay between epigenetic reprogramming, metabolic rewiring, and signaling pathway activation. In the epigenetic module, pre-training “closed” chromatin (modified by H3K9me3/H3K27me3) is converted to an “open” state (H3K4me3/H3K27ac modifications) via KDMs and TET2, following initial stimulation (e.g., β-glucan) activating NF-κB (through TLR2/4) and recruiting histone modifiers, enabling amplified cytokine (e.g., TNF, IL-6) transcription. In the metabolic module, initial stimulation drives enhanced glycolysis (HK, PFK, PKM2 activation), generating metabolites (acetyl-CoA, succinate) that link metabolism to epigenetics via mTOR; this enhances phagocytosis for pathogen clearance, while excessive activation triggers inflammation (e.g., NETosis). In the signaling module, initial stimulation activates TLR/PI3K-Akt-mTOR pathways, with amplified signaling upon secondary stimulation, collectively mediating either protective immune responses (green) or pro-inflammatory disease outcomes (red). Acetyl-CoA, Acetyl coenzyme A; Akt, Protein kinase B (PKB); ATP, Adenosine triphosphate; HK, Hexokinase; H3K27ac, Histone 3 lysine 27 acetylation; H3K27me3, Histone 3 lysine 27 trimethylation; H3K4me3, Histone 3 lysine 4 trimethylation; H3K9me3, Histone 3 lysine 9 trimethylation; IKK, Inhibitor of kappa B kinase; IL-1β, Interleukin-1 beta; IL, Interleukin; IRF, Interferon regulatory factor; KDM, Lysine demethylase; LPS, Lipopolysaccharide; mRNA, Messenger ribonucleic acid; mTOR, Mechanistic target of rapamycin; NETosis, Neutrophil extracellular trap formation; NF-κB, Nuclear factor kappa B; PFK, Phosphofructokinase; PI3K, Phosphoinositide 3-kinase; PKM2, Pyruvate kinase M2; ROS, Reactive oxygen species; TET2, Ten-eleven translocation methylcytosine dioxygenase 2; TLR2, Toll-like receptor 2; TLR4, Toll-like receptor 4; TNF, Tumor necrosis factor; TNF-α, Tumor necrosis factor-alpha.

The functional output of trained immunity has a dual nature. Protective functions include enhanced phagocytic capacity, improved pathogen clearance efficiency, and cross-protection against infections. However, when trained immunity is inappropriately activated or dysregulated by persistent endogenous or environmental signals, it can lead to pathogenic functions, driving the development of various chronic diseases. Excessive formation of NETs is a core pathological link.^53^ NETs, originally a mechanism for neutrophils to capture and kill pathogens, can, in the context of trained immunity, release large amounts of self-antigens upon overproduction, breaking immune tolerance. This is closely related to the development of autoimmune diseases like systemic lupus erythematosus and rheumatoid arthritis. Excessive inflammatory responses refer to tissue damage caused by abnormally trained innate immune cells continuously releasing high levels of inflammatory factors. Studies confirm this participates in the progression of atherosclerosis; trained monocytes and macrophages persistently produce factors like IL-1β in the vascular wall, promoting plaque formation and instability (Fig. 2).

### Systemic propagation of trained immunity

One of the most revolutionary features of trained immunity is that its effects can transcend the local site of the initial stimulus, achieving systemic propagation and long-term maintenance throughout the body. The core of this process lies in the transmission of inflammatory signals from a local infection or vaccination to the bone marrow—the birthplace of immune cells—via cytokines or metabolites in the circulatory system.^54^ Recent evidence suggests that intercellular communication mediated by membrane-associated mechanisms contributes to the propagation of trained immunity. Immune training-induced alterations in membrane organization, including changes in lipid composition and receptor distribution, have been proposed to facilitate the transmission of trained immune states between neighboring cells. This membrane-based mode of communication provides a conceptual framework for understanding how trained or exhaustion-associated innate immune states may spread within tissues and contribute to coordinated innate immune memory at the tissue level.^55^

The systemic effects of trained immunity are closely related to the functional reprogramming of hematopoietic stem and progenitor cells (HSPCs). Local inflammatory signals can induce persistent epigenetic reprogramming in bone marrow HSPCs. This alters normal hematopoietic differentiation, which depends on interactions between chromatin epigenetic structure and specific transcription factors,^56^ causing these cells and the myeloid cells they produce to become “pre-activated” at an early developmental stage, acquiring stronger pro-inflammatory potential. After these cells enter the peripheral blood and distribute to tissues throughout the body, they establish an innate immune defense layer for the organism in a state of heightened alert. When the body encounters any secondary infection, the immune system across various tissues can mount a faster, stronger response. This transforms a local inflammatory event into a lasting, broad-spectrum systemic immune enhancement state, even providing cross-protection against different pathogens.^1^

## The dual role of trained immunity in respiratory system diseases

On one hand, trained immunity can enhance the bactericidal activity and inflammatory cytokine secretion capacity of innate immune cells such as alveolar macrophages, providing the host with stronger anti-pathogen defense beyond the primary infection. This beneficial effect plays a role in combating reinfection by *Mycobacterium tuberculosis* (*M. tuberculosis*) or certain viral infections. However, inappropriately activated trained immunity may trigger amplified inflammatory responses and pathological tissue damage.^57^ LPS molecules found in the cell membranes of certain bacteria can induce excessive inflammation, leading to acute lung injury.^58^ Furthermore, a persistently hyperactive immune state can also lead to pathological damage. When trained immunity is continuously triggered by harmless environmental antigens (e.g., allergens) or self-antigens, it can cause over-activation of the immune system. This excessive inflammation resulting from inappropriate activation is a core pathological basis for many chronic respiratory diseases. Thus, while conferring protective benefits, the potential pathogenicity of trained immunity also profoundly influences the course of respiratory system diseases.

Dysfunction of the immune system in the respiratory tract can trigger immune imbalance, subsequently inducing various diseases. This imbalance primarily manifests as two common features: chronic inflammation and dysregulated immune memory. Chronic inflammation is characterized by the long-term presence of large numbers of activated immune cells (e.g., eosinophils, neutrophils, lymphocytes) within the airways. Histone modifications keep their pro-inflammatory genes in a persistently open state, leading to continuous release of various pro-inflammatory factors. This sustained inflammation directly damages lung tissue, causing airway structural remodeling (e.g., fibrosis, smooth muscle hyperplasia),^59^ excessive mucus secretion, and airway hyperresponsiveness. Allergic inflammation in asthma and smoke-related inflammation in COPD are classic examples of chronic inflammation. In asthma, overtrained innate immune cells amplify T helper 2 (Th2)-skewed inflammation, leading to sustained eosinophil recruitment, mucus hypersecretion, and airway remodeling.^60^^,^^61^ In COPD, chronic exposure to cigarette smoke and microbial products promotes maladaptive training of alveolar macrophages and neutrophils, resulting in persistent cytokine release, protease activation, and impaired tissue repair.^62^^,^^63^ These processes collectively contribute to progressive airflow limitation and emphysematous destruction.

**Fig. 3:**
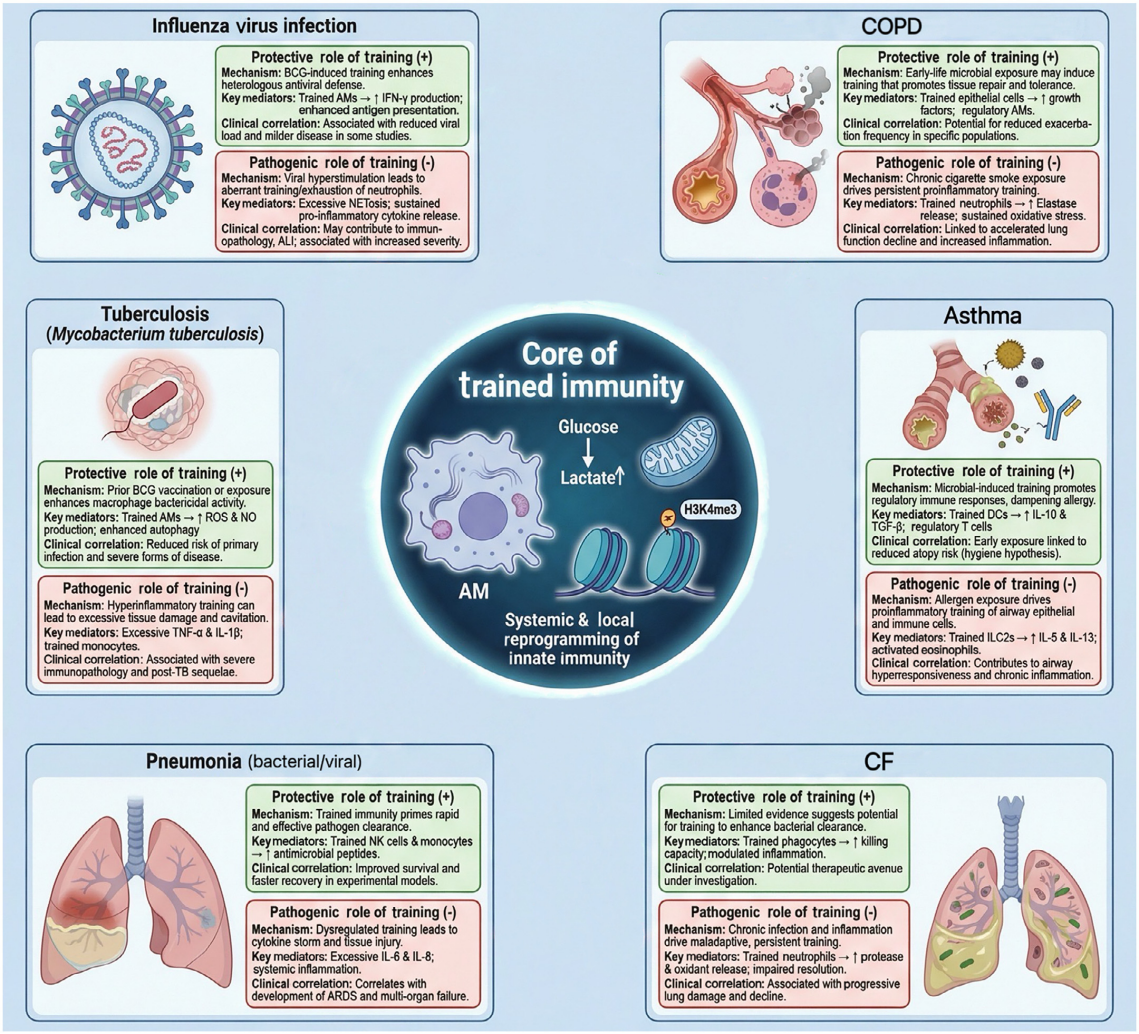
The role of trained immunity in different respiratory diseases. Core functions of trained immunity in AMs and its bidirectional roles in pulmonary diseases: Characterized by enhanced glucose metabolism and H3K4me3 epigenetic modification, trained immunity (via systemic and local innate immune reprogramming) exerts protective effects (green) in influenza virus infection, tuberculosis, pneumonia, cystic fibrosis (CF), COPD, and asthma—e.g., enhancing anti-bacterial/viral activity, and improving pathogen clearance and clinical outcomes. It also poses pathogenic risks (red) in influenza virus infection, tuberculosis, pneumonia, COPD, asthma and CF—e.g., excessive inflammation, tissue injury, and disease progression. Line thickness indicates the relative strength of association; protective roles center on boosted immune cell function and improved clinical prognosis, while pathogenic roles link to hyperactivation and chronic inflammation. ALI, Acute lung injury; AM, Alveolar macrophage; ARDS, Acute respiratory distress syndrome; BCG, Bacillus Calmette–Guérin; CF, Cystic fibrosis; COPD, Chronic obstructive pulmonary disease; DCs, Dendritic cells; H3K4me3, Histone 3 lysine 4 trimethylation; IFN-γ, Interferon-gamma; IL-1β, Interleukin-1 beta; IL-5, Interleukin-5; IL-6, Interleukin-6; IL-8, Interleukin-8; IL-10, Interleukin-10; IL-13, Interleukin-13; ILCs, Innate lymphoid cells; NK cells, Natural killer cells; NO, Nitric oxide; ROS, Reactive oxygen species; TB, Tuberculosis; TGF-β, Transforming growth factor-beta; TNF-α, Tumor necrosis factor-alpha.

Dysregulated immune memory can be further divided into two scenarios: over-reaction and hyporeactivity. Over-reaction refers to the immune system mistakenly identifying harmless substances (e.g., pollen, dust mites) as harmful and establishing a strong, erroneous immune memory. This leads to excessive and pathological immune responses upon re-exposure to such substances. Asthma is a typical example. IL-18 can potentiate Th2 cell immune responses, causing an imbalance in Th1/Th2 cell levels, which may exacerbate the condition.^64^ Conversely, hyporeactivity refers to a state in which prolonged or repeated immune stimulation induces functional exhaustion or tolerance of innate immune cells. In this condition, metabolic activity and pro-inflammatory cytokine production are diminished, and epigenetic landscapes may shift toward repressive chromatin states at inflammatory gene loci. Such maladaptive reprogramming compromises pathogen clearance and predisposes individuals to persistent or recurrent infections (Fig. 3).^65^

### Infectious respiratory diseases

#### Viral pneumonia

In the field of viral pneumonia, trained immunity exhibits a dual nature, with community-acquired pneumonia caused by influenza viruses and severe acute respiratory syndrome coronavirus 2 (SARS-CoV-2) (the virus responsible for COVID-19) being its primary contexts. Research by Yao et al^66^ demonstrates that IFN-γ produced by adaptive T cells can induce the formation of immunological memory in alveolar macrophages, leading to trained immunity against pulmonary bacterial infections. Pre-vaccination with vaccines such as BCG can also induce trained immunity, providing the host with cross-protection.^67^^,^^68^

However, overactivation of trained immunity may also yield detrimental outcomes. Following severe viral infections, lung neutrophils may undergo a pathological training process, resulting in an abnormally enhanced capacity to form NETs. The excessive release of these NET structures can trigger a “NETosis storm”, which not only fails to effectively clear the virus but also damages pulmonary vascular endothelial cells, exacerbating inflammatory leakage and lung tissue injury,^69^ thereby contributing to the development of acute respiratory distress syndrome (ARDS). Significant neutrophil proliferation has also been observed in COVID-19 patients.^70^ These findings collectively indicate that NETs produced during the immune training of neutrophils possess destructive effects on the lung tissue barrier.^71^

The balanced state of trained immunity is more fragile in the elderly population. Immunosenescence associated with aging leads to a gradual decline in the capacity of innate immune cells to undergo training, directly resulting in reduced immune protection efficacy post-vaccination.^72^ Consequently, even after completing vaccination, the elderly population remains at a higher risk of severe pneumonia, highlighting the critical importance of maintaining an appropriate balance of trained immunity for improving clinical prognosis. Clinical data indicate that fibromyalgia (FM)-like symptoms are prevalent among patients recovering from COVID-19, with obesity and male sex identified as risk factors for post-COVID FM development.^73^

Clinical observations in patients recovering from COVID-19 suggest that SARS-CoV-2 infection can induce persistent functional reprogramming of innate immune cells. Longitudinal analyses have shown that circulating monocytes from convalescent individuals display sustained transcriptional and epigenetic alterations, together with enhanced inflammatory responsiveness upon secondary stimulation.^74^^,^^75^ These trained immunity-like features have been proposed to contribute to prolonged inflammatory symptoms observed in a subset of patients after acute infection, highlighting the potential clinical relevance of dysregulated innate immune memory in post-COVID conditions.

#### Bacterial pneumonia

In the field of bacterial pneumonia, involving clinically common pathogenic strains such as *S. pneumoniae* and *Pseudomonas aeruginosa* (*P. aeruginosa*), trained immunity can provide broad-spectrum protection against bacterial infections.^76^ Trained alveolar macrophages (AMs) enhance their bacterial clearance capacity through metabolic reprogramming, specifically manifesting as a significant increase in glycolytic levels. This metabolic adjustment directly strengthens phagocytic function and bactericidal efficiency, enabling AMs to clear pathogens more rapidly and effectively during bacterial invasion.

Trained immunity can also act synergistically with antibiotics, offering assistance in the treatment of bacterial pneumonia. The trained metabolic mode can increase bacterial susceptibility to β-lactam antibiotics.^77^ This synergistic effect not only enhances the bacteriostatic or bactericidal efficacy of antibiotics but may also, to some extent, reduce the risk of resistant strain emergence, providing new insights for optimizing clinical therapeutic regimens.

Research has found that in patients with severe bacterial pneumonia, innate-like CD8+ T cells experience severe exhaustion, while monocyte-derived suppressor cells abnormally increase. These are key contributors to severe immunosuppression and excessive complement activation.^78^ This indicates that in severe bacterial pneumonia, trained immunity may face exhaustion issues, leading to a decline in the host’s sustained control over the bacteria. In cases of prolonged bacterial colonization in the lungs, the trained phenotype of AMs gradually diminishes, and their original functional advantages weaken accordingly. Concurrently, this can disrupt the inflammation–repair balance. This imbalanced state not only reduces host immunity but may also lead to non-healing lung tissue damage, becoming a significant factor in the difficulty of eradicating chronic infections and posing a new challenge for the development of subsequent intervention strategies.

#### Pulmonary tuberculosis

In the context of pulmonary tuberculosis and the immune regulation of *M. tuberculosis* infection, the BCG vaccine is a crucial vehicle for modulating trained immunity. Research by Kaufmann et al^79^ shows that intravenously injected BCG in mice can reach the bone marrow and reprogram hematopoietic stem cells, driving their differentiation toward the myeloid lineage. This process primarily generates monocytes, which enter tissues via the bloodstream and ultimately differentiate into macrophages, providing a potential cellular source for trained immunity. Meanwhile, clinical data on BCG revaccination corroborates its potential to regulate *M. tuberculosis* infection via trained immunity.^80^

Deficiencies in trained immune function significantly impact the disease progression of pulmonary tuberculosis, particularly related to the reactivation of latent infection. Triggering receptor expressed on myeloid cells 2 (TREM2) has been demonstrated to be associated with alveolar macrophage apoptosis.^81^ In the presence of *TREM2* gene mutations, the training capacity of AMs markedly decreases, and their inherent anti-mycobacterial activity weakens. This leads to a higher likelihood of latent *M. tuberculosis* overcoming immune control and progressing to active pulmonary tuberculosis. This mechanism provides a key target for understanding tuberculosis infection relapse and progression.

The development of novel vaccines based on the trained immunity mechanism is offering new directions for optimizing tuberculosis prevention strategies. Current exploration focuses on recombinant BCG vaccines. Compared to traditional BCG vaccines, these modified vaccines can induce longer-lasting immune protection in the host, effectively enhancing the duration and strength of defense against *M. tuberculosis* infection and addressing the shortcoming of insufficient effective antibody provision in adults.^82^ This offers a superior candidate strategy for future tuberculosis prevention and control (Table 1).

**Table 1:** Comparison of the characteristics of trained immunity in different respiratory diseases.

Disease type	Core trained immune cell(s)	Role of trained immunity	Key mechanisms	Potential training biomarkers	Clinical/translational association
Viral pneumonia (e.g., influenza, SARS-CoV-2)	AMs, monocyte-derived macrophages, neutrophils	Dual: • Protective: Heterologous antiviral defense. • Pathogenic: Immunopathology; cytokine storm.	• Epigenetic priming of antiviral genes (IFITMs, ISGs). • Trained macrophages enhance type I interferon response. • Enhanced glycolysis and altered mitochondrial metabolism. • Aberrant NETosis by neutrophils leading to tissue damage.	• ↑H3K4me3 at IFN-γ locus in peripheral monocytes. • Sustained expression of pro-inflammatory cytokines (IL-6, TNF-α). • Elevated plasma succinate/lactate. • Circulating NET components (cfDNA, MPO).	• BCG vaccination correlates with reduced severity of influenza/COVID-19 in some studies. • Monocyte training phenotype linked to severe COVID-19 outcomes. • Potential for trained immunity-based vaccines (e.g., BCG) against novel viruses.
Bacterial pneumonia (e.g., *S. pneumoniae*)	Alveolar macrophages, bone marrow myeloid progenitors	Predominantly protective: Enhanced bacterial clearance and sepsis resistance.	• H3K4me3/H3K27ac enrichment at promoters of pro-inflammatory genes (*TNF, IL6*). • Rewired cholesterol synthesis in hematopoietic stem and progenitor cells (HSPCs). • Enhanced phagocytosis and ROS production by trained macrophages.	• Elevated levels of H3K27ac at enhancer regions of pro-inflammatory genes. • Increased expression of glycolytic enzymes (HK2, PFKFB3). • Expanded populations of trained myeloid progenitors in bone marrow.	• Associated with reduced risk of secondary infections post-vaccination or mild primary infection. • Basis for the non-specific protective effects of β-glucan or BCG against bacterial sepsis.
Pulmonary tuberculosis	Lung-resident macrophages, monocytes, CD8+ T cells (innate-like memory)	Complex/Dual: • Protective: Enhanced containment of *M. tuberculosis*. • Pathogenic: Granuloma maintenance, excessive tissue damage.	• *M. tuberculosis*-induced epigenetic reprogramming in monocytes/macrophages (H3K4me3 at TNF, IL1β). • Metabolic shift towards aerobic glycolysis. • Induction of “innate memory” CD8+ T cells with rapid effector functions.	• Long-term cytokine recall response (e.g., TNF-α, IL-6) to mycobacterial antigens. • Specific histone modification signatures in circulating monocytes post-BCG vaccination. • Surface markers on trained innate-like lymphocytes (e.g., CD45RO+).	• BCG vaccination efficacy may be partly due to trained immunity. • Correlates with granuloma dynamics and disease progression/reactivation risk. • Potential target for host-directed therapies.
Asthma	Airway macrophages, type 2 innate lymphoid cells (ILC2s)	Dual: • Protective: Early-life microbial exposure mitigates allergic responses (“hygiene hypothesis”). • Pathogenic: Recurrent allergen exposure trains for exaggerated type 2 inflammation.	• Allergen-induced metabolic shift (glycolysis) in airway macrophages/ILC2s. • Histone modifications (H3K4me3) at enhancers of Th2 cytokine genes (*IL4, IL5, IL13*) in ILC2s. • Trained basophils/eosinophils with enhanced survival and mediator release.	• Epigenetic marks on Th2 loci in airway cells or peripheral ILC2s. • Persistently activated phenotype of airway macrophages post-allergen challenge. • Metabolic profile of airway lining fluid.	• Epidemiological link between farm/environmental exposures and reduced asthma risk. • May underpin severe, steroid-resistant asthma phenotypes. • Potential for epigenetic/metabolic modulators as novel therapeutics.
Chronic obstructive pulmonary disease (COPD)	Lung macrophages, sputum neutrophils	Predominantly pathogenic: Drives sustained inflammation, exacerbation frequency, and steroid resistance.	• Cigarette smoke/oxidant-induced epigenetic memory (DNA hypomethylation, H3K27ac) at pro-inflammatory loci (IL8, MMP9). • Trained airway neutrophils with enhanced NETosis and protease release. • Impaired resolution of inflammation due to altered macrophage metabolism.	• DNA methylation signatures in sputum macrophages/airway epithelium. • Persistently elevated inflammatory cytokines (IL-8, IL-1β) in airways. • Increased glycolytic enzyme expression in lung macrophages.	• Correlates with disease progression, frequency of acute exacerbations, and poor response to corticosteroids. • Trained immunity may explain “smoker’s memory” persisting after smoking cessation.

Lung fibrosis	Profibrotic macrophages, fibroblasts (non-immune, “memory-like”)	Predominantly pathogenic: Promotion of fibroblast activation and self-sustaining fibrosis.	• Mechanical stress/TGF-β-induced “memory” in fibroblasts via sustained H3K27ac at fibrotic gene promoters. • Profibrotic macrophage persistence driven by metabolic adaptation (e.g., increased PPP activity). • Trained immunity amplifies the feed-forward loop of injury and repair.	• Distinct histone modification patterns in lung fibroblasts from IPF patients. • Profibrotic gene expression signatures in monocyte-derived macrophages. • Metabolic markers in bronchoalveolar lavage fluid (BALF).	• Associated with rapid disease progression and poor response to standard therapy. • Suggests targeting epigenetic “memory” in fibroblasts/macrophages as a novel antifibrotic strategy.
Lung tumors	TAMs, MDSCs	Dual: • Protective: Therapy -induced (e.g., chemotherapy) M1-like polarization and tumoricidal activity. • Pathogenic: Tumor microenvironment educates pro-tumorigenic, M2-like polarization.	• Chemotherapy/immunotherapy induces immunogenic training of TAMs towards an M1 phenotype. • Tumor-derived lactate and metabolites drive epigenetic silencing in MDSCs (↑H3K9me3, DNA methylation). • Metabolic competition in TME shapes long-term myeloid cell function.	• Surface marker ratio (CD86/CD206) on TAMs. • Metabolic enzyme expression (iNOS *vs.* Arg1) in tumor-infiltrating myeloid cells. • Plasma levels of oncometabolites (e.g., lactate, succinate).	• M2-like TAM abundance correlates with poor prognosis and immunotherapy resistance. • Potential to “re-train” TAMs via metabolic/epigenetic modulators to enhance anti-tumor immunity. •Basis for combining chemotherapy with immune checkpoint inhibitors.

AMs, Alveolar macrophages; Arg1, Arginase 1; BCG, Bacillus Calmette–Guérin; CD45RO, Cluster of differentiation 45RO; CD8+ T cells, Cluster of differentiation 8 positive T cells; COVID-19, Coronavirus disease 2019; cfDNA, Cell‑free DNA; DNA, Deoxyribonucleic acid; H3K4me3, Histone H3 lysine 4 trimethylation; H3K9me3, Histone H3 lysine 9 trimethylation; H3K27ac, Histone 3 lysine 27 acetylation; HK2, Hexokinase 2; IFITMs, Interferon‑induced transmembrane proteins; IFN-γ, Interferon gamma; IL-1β, Interleukin-1β; IL-6, Interleukin-6; IL-8, Interleukin-8; IL-13, Interleukin-13; ILCs, Innate lymphoid cells; IL1B, Interleukin 1 beta; iNOS, Inducible nitric oxide synthase; IPF, Idiopathic pulmonary fibrosis; ISGs, Interferon-stimulated genes; LPS, Lipopolysaccharide; M1, Classically activated macrophage phenotype; M2, Alternatively activated macrophage phenotype; M-CSF, Macrophage colony-stimulating factor; MDSCs, Myeloid-derived suppressor cells; MMP9, Matrix metalloproteinase 9; MPO, Myeloperoxidase; NETosis, Neutrophil extracellular trap formation; NETs, Neutrophil extracellular traps; PFKFB3, 6-Phosphofructo-2-kinase/fructose-2:6-bisphosphatase 3; PPP, Pentose phosphate pathway; ROS, Reactive oxygen species; *S. pneumoniae, Streptococcus pneumoniae*; SARS-CoV-2, Severe acute respiratory syndrome coronavirus 2; TAMs, Tumor-associated macrophages; TGF-β, Transforming growth factor beta; Th2, T helper 2; TME, Tumor microenvironment; TNF, Tumor necrosis factor; TNF-α, Tumor necrosis factor-α.

### Chronic respiratory diseases

#### Asthma

In the pathological progression of asthma, the regulatory effect of trained immunity on airway hyperreactivity is particularly prominent. The abnormal activation of Th2 cells is a key factor leading to airway hyperreactivity, ultimately exacerbating typical symptoms in asthma patients such as airway spasm and constriction. The widely accepted view is that asthma is triggered by an imbalance between Th1 and Th2 immune pathways, particularly the excessive Th2-mediated inflammation.^83^ TLRs and NOD-like receptors (NLRs) expressed on epithelial cell surfaces can recognize PAMPs and induce immune responses. IL-25 secreted by the epithelium can lead to eosinophilia and induce Th2-type immune responses.^84^ Pre-stimulation with house dust mite (HDM) can induce a trained phenotype in dendritic cells (DCs). These trained DCs can initiate immune responses more efficiently, directly promoting the differentiation of Th2 cells.^85^

Aberrant training of ILC2s plays a critical role in the sustained development of airway inflammation in asthma. Under persistent stimulation by IL-25 or IL-33 in the airways, ILC2s can enter an overtrained state, significantly enhancing their capacity to secrete IL-5 and IL-13.^86^ IL-13, in particular, is a key cytokine driving pathogenic Th2 responses in allergic asthma. The over-secretion of these cytokines promotes airway mucus secretion and remodeling,^87^ creating a vicious cycle of inflammation. Regulatory T (Treg) cells, secreting IL-10 and transforming growth factor-beta (TGF-β), can suppress excessive inflammation and maintain immune balance.^88^

Both *in vitro* and *in vivo* experiments confirmed that upon ovalbumin (OVA) stimulation, wild-type AMs exhibited significantly increased *TLR2*/*HIF1A* expression, glycolytic activity, and polarization activation, whereas TLR2^-/-^ AMs showed no such changes. Loss of TLR2 in resident AMs ameliorated allergic airway inflammation (AAI) by inhibiting pyroptosis and oxidative stress, suggesting that the TLR2–HIF-1α–glycolysis axis in resident AMs may serve as a novel therapeutic target for asthma.^89^

Targeting the regulation of trained immunity has emerged as a potential direction for asthma treatment, with the mTOR pathway being a key target.^90^ Inhibiting mTOR pathway activity can effectively reverse the overtrained state of ILC2s, reducing their secretion of pro-inflammatory cytokines, thereby alleviating airway inflammation and symptoms such as wheezing and chest tightness in asthma patients. This provides a novel mechanism of action and therapeutic approach for the precise intervention of asthma.

#### Chronic obstructive pulmonary disease

In the pathological progression of COPD, the dysregulation of trained immunity is closely related to the onset and development of the disease, with cigarette smoking being a key factor inducing this abnormality.^91^ Studies have shown that during exposure to cigarette smoke extract (CSE), parkin RBR E3 ubiquitin protein ligase (PRKN) overexpression induces mitophagy. Experimental results suggest that PRKN may play a pivotal role in the pathogenesis of COPD by regulating mitophagy, and inducing PRKN expression may mitigate the progression of COPD by enhancing mitophagy, promoting the clearance of damaged mitochondria, reducing mitochondrial ROS accumulation, and restoring metabolic homeostasis in airway macrophages.^92^ CSE can directly lead to metabolic reprogramming disorders in AMs, manifesting as abnormally enhanced glycolytic pathway activity accompanied by a decline in phagocytic function. This imbalance between function and metabolism prevents AMs from effectively clearing harmful substances and pathogens from the airways, further aggravating airway damage and inflammation.

The interaction between trained immunity and inflammaging is a significant reason for the persistent inflammatory response in COPD patients. Research shows that histone lactylation (histone H4 lysine 12 lactylation, H4K12la) promotes the senescence of type II alveolar epithelial cells by modulating the CD38-nicotinamide adenine dinucleotide (NAD)+ signaling pathway, thereby exacerbating COPD progression.^93^ In COPD patients, significantly higher concentrations of IL-8 in sputum have been observed compared to asthma patients or controls,^94^ and IL-8 correlates with lung function in COPD patients. Histone lactylation (H4K12la) and CD38-NAD+ signaling mediate metabolic–epigenetic crosstalk, a core mechanism of trained immunity. Sustained IL-8 elevation in COPD reflects the long-term pro-inflammatory phenotype driven by trained immunity. These processes link epigenetic reprogramming, metabolic signaling, and persistent inflammation to the pathogenesis of COPD via trained immunity. In patient AMs, trimethylation of H3K4me3 is enriched in the promoter region of the C-X-C motif chemokine ligand 8 (*CXCL8*). This epigenetic alteration persistently activates CXCL8 expression, promoting the continuous release of inflammatory factors, establishing a state of chronic inflammation, and simultaneously accelerating immune cell senescence, further impairing the body’s ability to repair airway damage.

Biomarkers related to trained immunity show important value in the clinical assessment of COPD, potentially for predicting the risk of acute exacerbations. Clinical observations indicate that serum levels of IL-6 and the activity of PKM2 within AMs correlate with the severity of COPD and the probability of acute exacerbations. In the pathogenesis of COPD, airway remodeling is partly regulated through the inhibition of PKM2 activity by hedgehog-interacting protein (HHIP) within airway smooth muscle cells (ASMCs), which modulates glycolysis. This regulation can prevent excessive ASMC proliferation and help inhibit the progression of airway remodeling.^95^ Of note, PKM2-dependent glycolytic regulation represents a core metabolic mechanism underlying trained immunity. Although the above pathway functions in ASMCs, it highlights the conserved role of metabolic reprogramming in driving long-term cellular functional changes, which parallels the core characteristics of trained immunity and supports the integrated metabolic–pathological regulatory network in COPD. Detecting these markers can provide clinicians with references for prognosis judgment and the formulation of personalized intervention plans.

In COPD, clinical studies consistently demonstrate persistent activation of innate immune responses. Patients with stable COPD exhibit increased neutrophil and macrophage activation in the airways, accompanied by elevated production of pro-inflammatory mediators. This sustained innate immune activation correlates with disease severity and airway remodeling, suggesting that long-term functional alterations of innate immune cells may contribute to chronic inflammation and disease progression, resembling maladaptive forms of innate immune memory.^96^^,^^97^

#### Pulmonary fibrotic diseases

In the pathological process of pulmonary fibrosis, epithelial–mesenchymal transition (EMT) is considered to play a core regulatory role by reshaping the alveolar epithelial cell phenotype and activating fibroblasts.^98^ Trained immunity can drive EMT. Senescent alveolar epithelial cells release DAMPs, which can induce a trained phenotype in AMs. Trained AMs then secrete large quantities of TGF-β1. In the lungs, dysregulation of the TGF-β signaling pathway leads to cellular senescence and the release of pro-inflammatory mediators, forming a senescence-associated secretory phenotype (SASP).^99^ The cytokine TGF-β1 directly promotes fibroblast activation, driving collagen deposition and pulmonary tissue fibrosis progression.

Fatty acid metabolic reprogramming occurs in the state of pulmonary fibrosis. The abnormal metabolism in this process may lead to cellular dysfunction, affecting cell death and inflammatory responses, thereby promoting fibrosis progression.^100^ Fibroblasts in pulmonary fibrosis may also exist in a persistently activated state. Trained fibroblasts can maintain an activated phenotype by enhancing fatty acid oxidation. This metabolic feature is considered analogous to the metabolic reprogramming in trained immunity, although this classification requires more experimental validation.

Intervention strategies targeting trained immunity offer a potential direction for the treatment of pulmonary fibrosis. Changes in cellular metabolism within myofibroblasts may play a role in epigenetic modifications. The PPP is an important regulatory target within this context, which promotes the production of nucleotides and nicotinamide adenine dinucleotide phosphate (NADPH). NADPH is involved in regulating the redox state within trained AMs, influencing the activation of related signaling pathways, and thereby affecting the functional state of AMs. Targeting the PPP pathway using methods such as inhibitors of glucose-6-phosphate dehydrogenase can effectively inhibit the training process of AMs,^101^^,^^102^ reduce their secretion of pro-fibrotic cytokines, and mitigate the degree of pulmonary fibrosis, providing new therapeutic approaches for the clinical management of fibrotic lung diseases.

#### Lung cancer

Within the immune regulatory network of lung cancer, trained immunity may participate in both anti-tumor defense and act as a facilitator of tumor progression. The chemotherapeutic agent cisplatin can downregulate the expression of signal-regulatory protein α (SIRPα) and IL-6 in tumor-associated macrophages (TAMs), inducing their polarization toward an anti-tumorigenic M1 phenotype.^103^ This polarized macrophage population can recognize and attack tumor cells more efficiently while activating the host’s anti-tumor immune response, providing immunological support for enhancing chemotherapy efficacy. Macrophage polarization and functional reprogramming induced by cisplatin are closely associated with trained immunity. The long-term phenotypic and functional shift of tumor-associated macrophages toward an M1-like anti-tumor profile represents a persistent adaptive change in myeloid cells, which is consistent with the core definition of trained immunity—long-term enhanced immune responsiveness driven by epigenetic and metabolic reprogramming. This mechanism thus fits into the broader framework of trained immunity.

Conversely, if TAMs in the tumor microenvironment undergo excessive training, they can shift toward a pro-tumorigenic role. TAMs predominantly of the pro-tumor M2 phenotype secrete large amounts of cytokines such as vascular endothelial growth factor (VEGF), IL-10, and TGF-β,^104^ promoting tumor neovascularization and metastasis, and providing nutritional support for tumor growth. IL-10, in turn, promotes macrophage polarization toward the M2 type, suppresses the host’s anti-tumor immune response, aids tumor cells in achieving immune escape,^105^ and accelerates tumor invasion and metastasis.

The synergistic application of trained immunity and immunotherapy provides a new optimization direction for lung cancer treatment. Trained immunity inducers, such as β-glucan, can reverse resistance to anti-programmed cell death 1 (PD-1)/programmed death-ligand 1 (PD-L1) inhibitors.^106^ When combined with PD-1 inhibitors, they can further activate the host’s anti-tumor immune potential. This combination not only enhances the effect of PD-1 inhibitors in relieving the immunosuppressed state of the tumor,^107^ and redirecting M2 macrophages to an M1 state, but can also reverse the exhaustion of specific effector T cells within the tumor microenvironment, enhancing T cell killing activity, significantly improving overall treatment efficacy, and offering better therapeutic outcomes for lung cancer patients.

In addition, the memory-like properties of NK cells provide a novel direction for adoptive immunotherapy of lung cancer. Human cytokine-induced memory-like (CIML) NK cells, following brief preactivation with IL-12, IL-15, and IL-18, can form high-affinity IL-2 receptors (IL-2Rαβγ) through sustained CD25 expression, responding to picomolar concentrations of IL-2. This further enhances their proliferation, cytotoxicity, and IFN-γ secretion capabilities, with preferential expansion after adoptive transfer *in vivo*.^108^ The “preactivation and low-dose IL-2 maintenance” strategy can serve as a complementary regimen for lung cancer immunotherapy, particularly suitable for combination with immune checkpoint inhibitors.

#### Bronchiectasis

The typical pathological process of bronchiectasis manifests as a vicious cycle of recurrent infection, chronic inflammation, and airway damage. This process itself provides sustained stimuli for the activation and dysregulation of trained immunity. Studies show that there is a significant infiltration of neutrophils in the airways of patients with bronchiectasis.^109^ These neutrophils do not solely exert anti-infective functions; their function is reshaped in the long-term inflammatory environment. Over 80% of airway neutrophils express lectin‑type oxidized LDL receptor 1 (LOX-1) (a specific marker for polymorphonuclear myeloid-derived suppressor cells [PMN-MDSCs]), transforming into polymorphonuclear myeloid-derived suppressor cells (PMN-MDSCs) with immunosuppressive functions. These cells suppress T cell proliferation by secreting large amounts of arginase 1 (ARG-1), thereby creating a local immunosuppressive microenvironment in the airways.^110^ This phenomenon is particularly pronounced in patients during acute exacerbations, and the proportion of PMN-MDSCs in sputum is negatively correlated with the time to the next exacerbation. Pattern recognition receptors (e.g., TLR2, TLR4) on innate immune cells in patients are persistently activated, initiating inflammatory responses, recruiting more immune cells, regulating downstream signaling pathways, and participating in the “training-like” remodeling of immune cells, exacerbating immune dysregulation and airway damage.

External interventions can positively regulate innate immune function through training, offering new directions for the treatment of bronchiectasis. For example, regular exercise, as a non-infectious immune stimulus, can train the body’s innate immune system, enhancing immune cell activity and respiratory defense capabilities, thereby reducing the frequency of acute exacerbations. This protective effect aligns with the characteristics of trained immunity, such as exercise-induced metabolic reprogramming of immune cells and enhanced anti-infective capacity. Clinical studies show that stable patients experience improved exercise tolerance and quality of life after 6 to 8 weeks of exercise training, with a reduced risk of acute exacerbations over a 12-month period.^111^ Furthermore, antibiotic treatment can significantly reduce the proportion of PMN-MDSCs in the patient’s airway and blood,^110^ indirectly reversing the immunosuppression caused by abnormal immune training. Other approaches, such as fecal microbiota transplantation, probiotic supplementation, or low-dose immunomodulators (e.g., thymosin), can also achieve “training-like” regulation of bronchiectasis by modulating the immune–microbiome axis or directly enhancing immune function,^109^ providing diversified immunomodulatory strategies for disease management (Table 1).

## Intervention strategies for respiratory diseases based on trained immunity

**Fig. 4:**
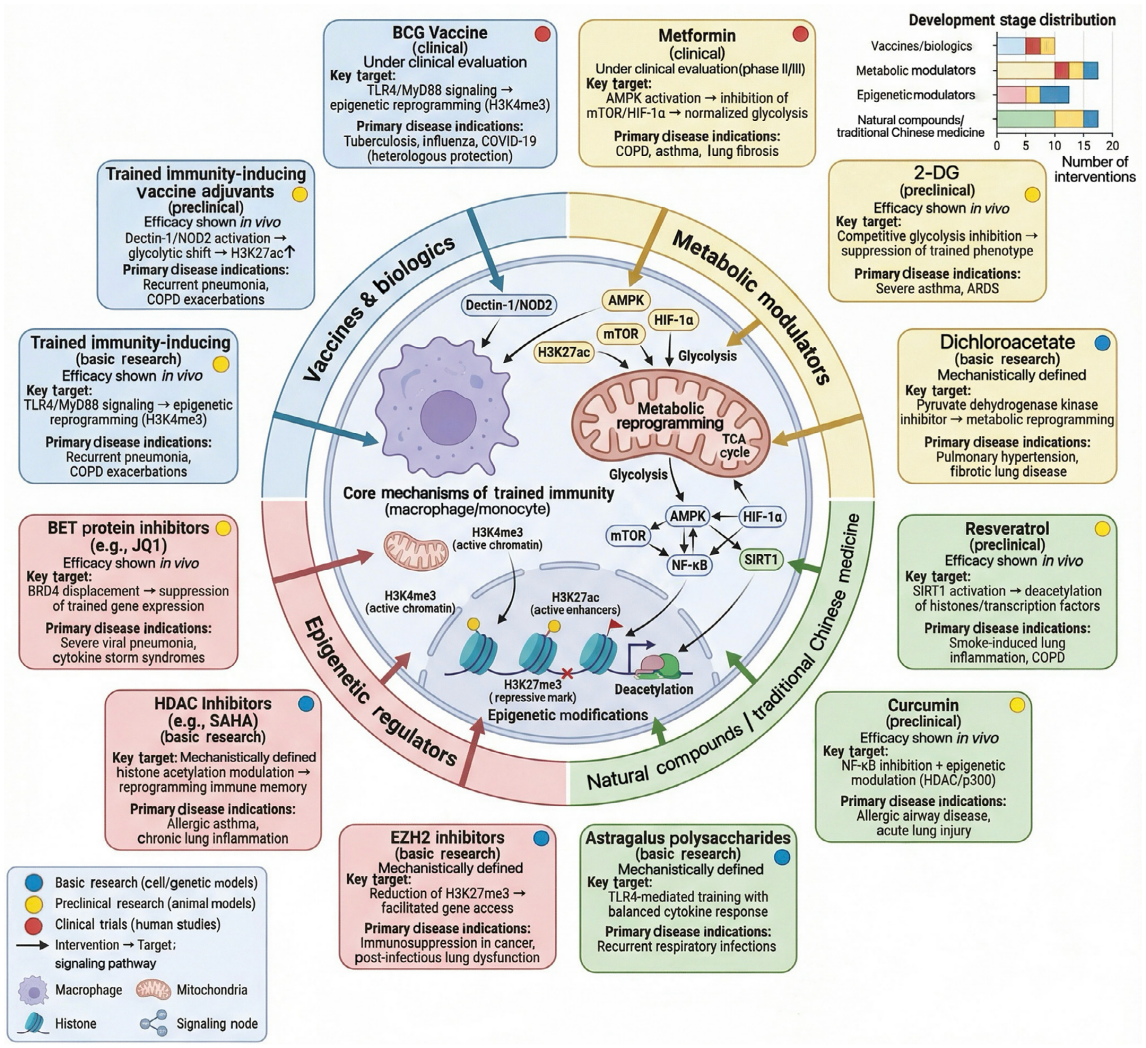
Targets for intervention strategies in respiratory diseases based on trained immunity. Potential therapeutic interventions targeting trained immunity pathways in respiratory diseases: Centered on epigenetic modifications (e.g., H3K4me3, H3K27ac) and metabolic reprogramming (glycolysis, mTOR/AMPK signaling) in macrophages/monocytes, interventions are categorized into vaccines/biologics, metabolic modulators, epigenetic regulators, and natural compounds/Chinese medicine. Vaccines (e.g., BCG) enable broad protection via epigenetic reprogramming; metabolic modulators (e.g., metformin) target glycolysis; epigenetic regulators (e.g., BET inhibitors) modulate chromatin activity; natural compounds (e.g., curcumin) co-regulate epigenetic and metabolic processes. These strategies are at distinct development stages (basic research → preclinical → clinical trials), primarily targeting diseases like COPD, asthma, and pulmonary fibrosis. In the top-right bar chart, red, yellow, blue, and light-colored areas represent interventions at the clinical, preclinical, basic research, and hypothesis stages, respectively. Two promising strategies are proposed: BCG for broad respiratory infection protection, and metabolic reprogramming drugs to normalize dysregulated glycolysis in COPD. ALI, Acute lung injury; AMPK, AMP‑activated protein kinase; ARDS, Acute respiratory distress syndrome; BRD4, Bromodomain‑containing protein 4; BCG, Bacillus Calmette–Guérin; BET, Bromodomain and extraterminal domain proteins; COPD, Chronic obstructive pulmonary disease; COVID-19, Coronavirus disease 2019; 2-DG, 2‑Deoxy‑D‑glucose; EZH2, Enhancer of zeste homolog 2; H3K4me3, Histone 3 lysine 4 trimethylation; H3K27ac, Histone H3 lysine 27 acetylation; HDAC, Histone deacetylase; HIF-1α, Hypoxia-inducible factor-1 alpha; mTOR, Mechanistic target of rapamycin; MyD88, Myeloid differentiation primary response 88; NF-κB, Nuclear factor-κB; NOD2, Nucleotide‑binding oligomerization domain‑containing protein 2; SAHA, Suberoylanilide hydroxamic acid; SIRT1, Sirtuin 1; TCA, Tricarboxylic acid; TGF-β, Transforming growth factor-β; TLR4, Toll‑like receptor 4.

Based on the core principle of trained immunity, which is inducing long-lasting and broad-spectrum functional enhancement of the innate immune system through appropriate stimulation—researchers are actively exploring its translation into innovative prevention and treatment strategies for respiratory diseases. These interventions aim to “re-educate” or reprogram immune cells, thereby enhancing the host’s defense capacity against infections, allergies, tumors, and other conditions. In addition to stimulus-based induction strategies, increasing attention has been directed toward therapeutic approaches that exploit functionally reprogrammed innate immune cells themselves. Recent studies demonstrate that monocytes subjected to trained or exhaustion-associated reprogramming acquire stable functional phenotypes characterized by altered cytokine production, metabolic profiles, and inflammatory potential, which can be harnessed to modulate disease outcomes. Similarly, neutrophils are increasingly recognized as capable of undergoing sustained functional reprogramming, enabling them to exert prolonged immunomodulatory effects beyond their traditionally perceived short lifespan.^112^ These reprogrammed innate immune cells can shape local inflammatory environments in the respiratory tract, influencing host defense, tissue damage, and repair processes.^45^ Collectively, these findings support the concept that targeting or leveraging trained monocytes and neutrophils represents a promising cell-centered intervention strategy in respiratory diseases. Below, we will systematically sort out the diversified intervention approaches based on this mechanism (Fig. 4).

### Vaccine-induced trained immunity

#### Non-specific vaccines

BCG and its derivatives, such as BCG polysaccharide nucleic acid, belong to a class of non-specific vaccines. They can increase the expression of key factors like TLR2 and TLR4 in the host’s Toll-like receptor pathway,^113^ activate downstream signaling pathways, and induce epigenetic reprogramming in monocytes and alveolar macrophages, thereby establishing trained immunity. In clinical applications, BCG demonstrates significant protective effects against influenza virus infection in the elderly. A double-blind, randomized clinical trial by Giamarellos-Bourboulis et al^114^ showed that after vaccinating elderly patients with BCG, the incidence of new infections decreased from 42.3% in the placebo group to 25.0% in the BCG-vaccinated group. Most of this protective effect was attributed to respiratory infections likely caused by viruses, and the time to first infection was significantly delayed in the BCG group, highlighting its important public health potential. With the deepening understanding of trained immunity mechanisms, progress has also been made in the development of novel non-specific vaccines: Recombinant BCG vaccines come in various types, all sharing the commonality of being genetically engineered modifications of the original BCG strain to enhance its immune functions or confer new properties. Research by Chiwala et al^115^ showed that a novel recombinant drug-resistant BCG (RdrBCG) strain, obtained by integrating the *Ag85B* and *Rv2608* genes into the genome of drug-resistant wild-type BCG through genetic engineering, can provide targeted adjunctive treatment for drug-resistant tuberculosis. In addition to BCG, β-glucan derived from *Candida albicans* is an important nonspecific immune activator that induces trained immunity in monocytes and macrophages via the Dectin-1/CARD9 signaling pathway in a mouse model of *S. pneumoniae* challenge, significantly enhancing bacterial clearance.^116^^,^^117^ Recent randomized clinical trials further support the role of BCG-induced trained immunity in reducing respiratory infection incidence and severity, particularly among elderly populations and individuals with comorbidities, although disease-specific benefits remain under active investigation.^114^^,^^118^

#### Enhancement of specific vaccine efficacy via trained immunity

Research has found that combining traditional specific vaccines with trained immunity inducers or vaccine adjuvants can enhance and prolong vaccine protection. For example, when influenza vaccines are co-administered with aluminum salts or TLR7 agonists,^119^ the duration of specific immune protection against influenza virus in recipients is extended. Studies have shown that SARS-CoV-2 messenger RNA (mRNA) vaccination establishes H3K27ac at promoters in human monocyte-derived macrophages, demonstrating the induction of highly dynamic and sustained training effects in innate immune cells.^120^

### Metabolic modulators

#### Glycolysis inhibitors/activators

Aberrant trained immunity can cause pathological damage. Glycolysis inhibitors such as 2-deoxy-D-glucose (2-DG) can exert anti-inflammatory effects to ameliorate such damage. Studies have shown that 2-DG blocks abnormal metabolic processes by inhibiting ILC2 function, thereby eliminating allergic airway inflammation.^121^ Furthermore, 2-DG significantly inhibits the LPS-induced increase in IL-1β levels in M1-polarized macrophages,^122^ effectively reducing excessive inflammation driven by pro-inflammatory responses.

Appropriately activating relevant metabolic pathways can enhance host immune defense and induce beneficial trained immunity. In clinical studies of COVID-19, metformin treatment is associated with reductions in disease severity and mortality.^123^ Metformin can be used to activate AMPK,^124^^,^^125^ promoting the training of alveolar macrophages and enhancing their phagocytic and clearance capabilities against pathogens like *S. pneumoniae*. AMPK exerts context-dependent regulatory effects on immune training. As a metabolic checkpoint, AMPK generally restricts excessive inflammatory activation to maintain immune homeostasis. However, in alveolar macrophages, metformin-mediated AMPK activation supports non-inflammatory, host defense-oriented trained immunity characterized by enhanced phagocytosis and microbial clearance, rather than pro-inflammatory pathological training. This indicates that AMPK can selectively promote protective trained immunity while restraining aberrant inflammatory reprogramming, consistent with its role as a negative regulator of excessive immune activation. In addition to AMPK, other signaling pathways such as p38 MAPK also play critical roles in host defense. van den Blink et al^126^ tested the effects of p38 MAPK inhibition in various cell types and different mouse models of infectious diseases. They observed increased cytokine production and significantly reduced bacterial clearance in mouse models of pneumococcal pneumonia and tuberculosis, suggesting a role for MAPK signaling in pneumococcal pneumonia.^127^ These findings indicate that p38 MAPK signaling is required for effective antibacterial defense, and that indiscriminate inhibition of this pathway may impair pathogen clearance. Therefore, therapeutic strategies targeting MAPK signaling must carefully balance inflammatory control with preservation of host antimicrobial capacity.

#### Tricarboxylic acid cycle intermediates

Intermediate metabolites of the TCA cycle, such as succinate, citrate, and α-ketoglutarate, can act as key signaling molecules in trained immunity. Research by Tannahill et al^122^ demonstrated that chronic activation of macrophages with LPS leads to an intracellular increase in succinate. Here, succinate acts as an endogenous danger signal stabilizing HIF-1α, thereby specifically regulating the gene expression of *Il1b* and other HIF-1α-dependent genes, and leading to protein succinylation. Moreover, exogenous succinate can enhance LPS-induced mRNA expression of HIF-1α target genes (e.g., *Phd3/Egln3*), regulating the metabolic reprogramming process in macrophage trained immunity. From a therapeutic perspective, targeted modulation of the succinate–HIF-1α pathway may enhance host defense during acute infection; however, sustained succinate accumulation should be carefully controlled to avoid excessive inflammatory activation.

Citrate, as the initial intermediate of the TCA cycle, influences macrophage energy metabolism (e.g., oxidative phosphorylation) and immune function (e.g., inflammatory cytokine secretion) through its level fluctuations.^128^ Citrate metabolism is a key link in the LPS-mediated activation of the NF-κB pathway in macrophages. LPS induces the transport of citrate from mitochondria to the cytosol via the mitochondrial citrate carrier through TLR4 signaling. In the cytosol, citrate is cleaved into acetyl-CoA, which cooperatively activates the NF-κB pathway, promoting the expression of pro-inflammatory factors like TNF-α and cyclooxygenase-2 (COX2) (Table 2).^129^ Therapeutically, inhibition of ATP citrate lyase (ACLY) to limit cytosolic acetyl-CoA production may attenuate NF-κB activation and excessive pro-inflammatory cytokine release, representing a potential strategy for chronic inflammatory airway diseases.

**Table 2:** Research progress statistics and comparison of trained immunity intervention strategies in respiratory diseases.

Disease type	Intervention category	Specific intervention	Primary target	Disease model/population	Research stage	Key findings/core outcome	Representative references
Viral pneumonia (e.g., Influenza)	Vaccine	BCG (intravenous *vs*. intradermal)	TLR2/4, MyD88, hematopoietic stem/progenitor cells	Mouse influenza model; human observational studies	Preclinical/clinical (epidemiological)	iv BCG protects against heterologous influenza via trained HSPCs generating altered monocytes; id BCG shows variable effects.	^49^^,^^68^
Viral pneumonia (COVID-19)	Metabolic modulator	Metformin	AMPK, mTOR, glycolysis	Human patients (retrospective & RCTs)	Clinical trial (Phase III/IV)	Associated with reduced severity and mortality; proposed mechanism includes blunting of maladaptive trained monocyte responses.	^73^^,^^123^
Bacterial pneumonia (*S. pneumoniae*)	Vaccine/biologic	β-glucan (from Candida)	Dectin-1, CARD9	Mouse *S. pneumoniae* challenge model	Preclinical	Systemic β-glucan induces trained immunity in monocytes/macrophages, enhancing bacterial clearance and survival.	^116^^,^^117^
Pulmonary tuberculosis	Vaccine	BCG revaccination	Innate immune memory in monocytes/macrophages	Non-human primates; human adolescents (clinical trial)	Clinical trial (Phase II)	Revaccination boosts non-specific protective monocyte responses (enhanced IL-1β, TNF-α) alongside specific immunity.	^54^^,^^80^
Asthma	Metabolic modulator	2-DG	Glycolysis (HK, PFK)	Mouse model of HDM-induced allergic asthma	Preclinical	Glycolysis inhibitors (e.g., 2-DG) can suppress aberrant trained immunity-like responses in airway macrophages by blocking training-dependent metabolic reprogramming, thereby alleviating eosinophilic inflammation and AHR.	^31^
Asthma	Natural compound	Resveratrol	SIRT1, HIF-1α, histone acetylation	Mouse OVA-induced asthma model; human asthmatic PBMCs (*in vitro*)	Preclinical/basic research	Attenuates airway inflammation and remodeling; *in vitro*, it modulates histone acetylation and cytokine production in trained monocytes.	^89^
COPD	Metabolic modulator	Dichloroacetate	Pyruvate dehydrogenase kinase → metabolic shift	Human PBMCs from COPD patients (*ex vivo*); mouse CS model	Basic research/preclinical	Reverses the hyper-glycolytic state of COPD monocytes, normalizing excessive LPS-induced cytokine production.	^92^
Lung tumors	Vaccine/cellular therapy	Trained NK cell therapy	Cytotoxicity receptors, metabolic pathways	Mouse melanoma lung metastasis model; human NK cells (*in vitro*)	Preclinical/basic research	Cytokine pre-conditioning (IL-12/15/18) induces a trained phenotype in NK cells with enhanced recall responses and anti-metastatic activity.	^108^

AHR, Airway hyperresponsiveness; AMPK, Adenosine monophosphate-activated protein kinase; BCG, Bacillus Calmette–Guérin; CARD9, Caspase recruitment domain-containing protein 9; COPD, Chronic obstructive pulmonary disease; COVID-19, Coronavirus disease 2019; CS, Cigarette smoke; 2-DG, 2-Deoxy-D-glucose; HDM, House dust mite; HIF-1α, Hypoxia-inducible factor 1-alpha; HK, Hexokinase; HSPCs, Hematopoietic stem/progenitor cells; id, Intradermal; IL-1β, Interleukin-1 beta; iv, Intravenous; LPS, Lipopolysaccharide; mTOR, Mechanistic target of rapamycin; MyD88, Myeloid differentiation primary response protein 88; NK cells, Natural killer cells; OVA, Ovalbumin; PBMCs, Peripheral blood mononuclear cells; PFK, Phosphofructokinase; RCTs, Randomized controlled trials; SIRT1, Sirtuin 1; *S. pneumoniae, Streptococcus pneumoniae*; TLR2, Toll-like receptor 2; TLR4, Toll-like receptor 4; TNF-α, Tumor necrosis factor-α.

### Traditional Chinese medicine and natural products

#### Polysaccharide components

Astragalus polysaccharide (APS), derived from the traditional qi-tonifying herb *Astragalus membranaceus*, possesses antioxidant properties. Research by Qiu et al^130^ found that APS enhances the phagocytic capacity and ameliorates the oxidative stress state of alveolar macrophages in a COPD mouse model, exerting a protective effect. Polysaccharides from *Ganoderma lucidum* (Reishi mushroom) demonstrate application value in inhibiting pathological trained immunity. In idiopathic pulmonary fibrosis (IPF), alveolar macrophages may be driven towards a pro-fibrotic phenotype by aberrant signals, promoting disease progression.^131^ Ganoderma polysaccharides can restore mTOR pathway regulation to suppress this detrimental training process,^132^ reverse macrophage metabolic abnormalities, and hinder their function in promoting collagen deposition and fibrosis. This provides a natural metabolic-immunomodulatory strategy for intervening in pulmonary fibrosis progression.

#### Other active components

Curcumin has been observed to exert protective effects in a rat COPD model. Zhang et al^133^ suggest this effect may be achieved by inhibiting endoplasmic reticulum stress in alveolar epithelial cells. Curcumin can influence epigenetic mechanisms such as histone modifications and DNA methylation,^134^ which may reverse the aberrant trained state of alveolar macrophages and alleviate airway inflammatory responses. Resveratrol is an activator of the deacetylase SIRT1.^135^ SIRT1, as a key aging-regulatory protein, aids in restoring metabolic and epigenetic plasticity in senescent immune cells upon activation, partially reversing the decline in trained immune function associated with organismal aging.

### Cell therapy and gene editing

#### Infusion of trained immune cells

For immunocompromised patients or those with drug-resistant infections, therapies involving the *ex vivo* induction and subsequent reinfusion of trained immune cells can be employed. Examples include biological DC-CIK cell therapy or the *ex vivo* pre-treatment of monocytes/macrophages using BCG or specific cytokine cocktails.^23^ After completing antibacterial training, these cells are reinfused into the patient, significantly enhancing pulmonary pathogen clearance capacity.

Immune training strategies mediated by exosomes can circumvent the potential risks associated with live cell transplantation. Mesenchymal stem cell (MSC)-derived exosomes carry various bioactive molecules. Clinically validated, human MSC-derived exosomes exhibit therapeutic effects and demonstrate immunosuppressive activity in animal disease models.^136^ Research finds that engineered and pre-conditioned MSC exosomes can significantly attenuate lung inflammation by acting on the Kruppel-like factor 5 (KLF5)/NF-κB signaling pathway through their enriched miR-145-5p,^137^ indicating their capacity to regulate downstream signaling pathways, induce lasting epigenetic and metabolic remodeling in innate immune cells, and generate protective trained immunity.

#### Gene editing for regulating trained immunity

Gene editing technologies enable precise modulation of trained immunity. Clustered regularly interspaced short palindromic repeats (CRISPR)–CRISPR associated protein 9 (Cas9)-mediated editing of the *TNFAIP3* gene, which inhibits NF-κB signaling pathway activity to negatively regulate immune responses,^138^ leads to the hypothesis that alveolar macrophages edited in this way would exhibit enhanced pro-inflammatory cytokine production and more efficient bacterial clearance capabilities when encountering *M. tuberculosis*.

Epigenetic editing technologies allow for the programmable modification of the memory state of immune cells without altering the DNA sequence. The fusion of catalytically dead CRISPR-associated protein 9 (dCas9) with epigenetic modifying enzymes (e.g., p300, also known as lysine acetyltransferase 3B [KAT3B]) has been validated as feasible and shown to act on gene promoters.^139^ One study utilized a dCas9 fusion system with epigenetic enzymes (ten-eleven translocation methylcytosine dioxygenase 1 [TET1]/DNA methyltransferase 3A [DNMT3A]) to precisely edit the DNA methylation state at the *IL1RN* gene promoter in human myeloid cells, stably altering the cellular response pattern to inflammatory stimuli.^140^ Lysine acetyltransferase 3A (KAT3A)/p300 family proteins and the histone acetylation they catalyze are key regulators of pro-inflammatory cytokine expression, such as IL-6.^141^ Fusing catalytically dead dCas9 with specific epigenetic modifying enzymes like KAT3A to target the promoters of key pro-inflammatory genes (e.g., *IL6*) and enhance repressive histone marks could suppress the overtrained inflammatory response in asthma.

### Lifestyle interventions

Long-term adherence to regular aerobic exercise can activate the AMPK signaling pathway,^142^ positively regulating the training process of alveolar macrophages and enhancing pulmonary anti-infective capacity. Dietary fiber, metabolized by gut microbiota to produce short-chain fatty acids, can influence the dynamic balance of trained immunity via the gut–immune axis, helping to reduce inflammation and protect against chronic diseases.^143^ Vitamin D can inhibit the expression of interleukin IL-6, IL-8, and tumor necrosis factor TNF-α,^144^ directly acting on immune cell signaling pathways to participate in regulating trained immunity balance. Sleep is a fundamental physiological process for maintaining bodily homeostasis and exerts a significant influence on the proper functioning of trained immunity. Chronic sleep deprivation leads to a marked decline in trained immune function and can trigger systemic inflammation akin to a cytokine storm (Table 2).^145^

### Major concern of trained immunity-based interventions

While multiple trained immunity-based intervention strategies have shown promising immunomodulatory effects in experimental settings, their clinical efficacy in respiratory diseases remains heterogeneous and context-dependent. Clinical studies evaluating BCG vaccination have demonstrated reduced incidence and severity of respiratory infections, particularly viral infections, in elderly and high-risk populations.^146^ However, the magnitude of protection varies substantially across cohorts and disease settings. Moreover, trained immunity induction is not universally beneficial, as excessive or prolonged activation may exacerbate chronic airway inflammation or tissue injury, especially in patients with asthma or COPD. Metabolic modulators targeting glycolysis or mTOR signaling pathways exhibit potential anti-inflammatory or immune-enhancing effects, yet their systemic metabolic impact raises concerns regarding long-term safety, off-target effects, and patient tolerance. Similarly, natural products and immune adjuvants often lack standardized dosing and robust clinical validation. These findings highlight key translational challenges for trained immunity-based therapies in respiratory diseases, including inter-individual variability, difficulty in defining optimal training intensity, and the risk of immune overactivation. Rigorous clinical trials incorporating efficacy endpoints, safety profiles, and immune monitoring are therefore essential before widespread clinical implementation.

### Emerging technologies and future directions

Research in the field of trained immunity is evolving from mechanistic exploration toward clinical application, propelled by the continuous emergence of new technologies: Single-cell sequencing technologies can clearly capture the dynamic metabolic reprogramming and epigenetic modifications in immune cells during the training process, clarify the activation patterns of specific genetic loci, and provide target guidance for subsequent interventions. You et al^147^ in their study comparing chromatin accessibility landscapes and T cell receptors in immune cells from COVID-19 convalescent individuals and healthy controls, observed a global remodeling of chromatin accessibility landscapes in the convalescent individuals, indicating the establishment of trained immunity. Organoid technology constructs *in vitro* three-dimensional models simulating trained immunity, enabling more accurate recapitulation of the *in vivo* micro-physiological environment and immunopathological processes.^148^ Utilizing co-culture systems of intestinal or pulmonary organoids with immune cells allows direct observation of the regulatory effects of different stimuli on trained immunity, avoiding interference from individual variations present in traditional animal experiments.^149^

The future development of trained immunity research relies on the interdisciplinary application and clinical translation of technologies. The current core challenges lie in achieving precise induction and timely reversal of the trained state in immune cells at specific body sites *in vivo*, as well as identifying cellular epigenetic markers or metabolic signatures to accurately assess an individual’s immune training level. Solving these problems is key to advancing its clinical translation. The development of novel immunomodulators,^150^^,^^151^ the formulation of personalized trained immunity regimens, and the organic integration of trained immunity strategies with traditional vaccine technologies are progressively transforming trained immunity from a theoretical concept into an intervention with clinical application value.^152^ This will open new avenues for prevention and treatment in fields such as infection control, cancer immunotherapy, and chronic inflammation management.

## Challenges and prospects

While trained immunity-based prevention and treatment strategies for respiratory diseases have provided an innovative direction to break through the limitations of traditional approaches and demonstrated broad application potential, they still face numerous bottlenecks and uncertainties that urgently need to be addressed in the entire chain of development from basic mechanism elucidation to practical clinical translation. Systematically sorting out and conducting in-depth analysis of the current limitations and challenges is a crucial prerequisite for accurately identifying research directions and promoting the steady development of this field. Below, we will first focus on the core issues and challenges at the current stage for in-depth discussions, and on this basis, further look forward to the future development paths and breakthrough directions.

### Current limitations and challenges

In trained immunity research, achieving specific regulation remains a core challenge. The difficulty lies in precisely controlling the intensity of training to avoid tissue damage caused by overtraining. While current mainstream interventions can effectively activate trained immunity, the boundary between “effective activation” and “over-activation” is still poorly defined.

The lungs are a critical effector site for trained immunity, and significant heterogeneity exists in the training mechanisms of different lung immune cells. The training process of alveolar macrophages primarily depends on metabolic reprogramming,^153^ for example, through AMPK pathway activation leading to a shift in energy metabolism patterns. In contrast, neutrophil training is more closely associated with epigenetic modifications, where changes in histone acetylation levels at specific gene loci directly impact their bactericidal activity.^154^ This cell-specific mechanistic diversity suggests that trained immunity interventions for lung diseases require cell-targeted strategies rather than a uniform activation approach.

The translation of trained immunity from basic research to clinical application still faces two major bottlenecks. First, there is a current lack of quantitative biomarkers for clinical detection of trained immunity. Existing evaluation methods mostly rely on functional assays of immune cells, which are operationally complex, difficult to standardize, and unable to rapidly and accurately reflect an individual’s trained immunity status. Second, the long-term safety of most intervention strategies remains unclear due to insufficient data. For instance, whether prolonged use of TLR agonist-type training adjuvants might lead to immune cell exhaustion or induce autoimmune reactions has not yet reached a definitive conclusion.^155^^,^^156^ These unknown factors significantly limit the practical application of trained immunity in fields such as chronic disease prevention/management and vaccine efficacy enhancement.

### Future prospects

#### Mechanism elucidation

Single-cell multi-omics technologies provide a novel perspective for deciphering the dynamic trajectories of lung immune cell training in mechanism elucidation research.^157^^,^^158^ Spatial transcriptomics technology can clearly reveal gene expression differences in cells across distinct lung regions.^159^ These varied microenvironments lead immune cells residing in different areas to develop specific trained immunity patterns. For example, macrophages near airway epithelium tend to express inflammation-related training markers, while alveolar macrophages exhibit training features primarily related to phagocytic function.^160^ This understanding aids in the precise targeting and regulation of lung trained immunity.

The interaction between trained immunity and the gut–lung axis is another important direction in mechanistic research. The core lies in uncovering the mechanisms by which gut microbiota metabolites remotely regulate pulmonary trained immunity. Metabolites produced by gut microbiota, such as short-chain fatty acids and tryptophan metabolites, can reach the lungs via the bloodstream. However, how these substances precisely act on the training processes of lung immune cells requires further in-depth exploration.

Research on the impact of aging on trained immunity and corresponding intervention strategies is becoming a key field for addressing immunosenescence. Current studies not only focus on the molecular mechanisms of aging-associated decline in trained immunity (e.g., mitochondrial dysfunction, epigenetic dysregulation), but are also dedicated to developing targeted reversal strategies. Examples include improving immune cell metabolic states by supplementing nutrients like vitamin D and coenzyme Q10,^161^ or using low-intensity endurance training to stimulate and reshape the training capacity of senescent immune cells.^162^ The exploration of such strategies will provide new avenues for maintaining trained immune function and improving health outcomes in the elderly population.

#### Technological innovation

In the clinical translation of trained immunity, the development of non-invasive detection technologies is key to overcoming assessment bottlenecks. Exhaled metabolomics and circulating microRNA biomarkers are current focal points. Traditional assessment of trained immunity relies on invasive cellular functional assays, making dynamic, repeated monitoring difficult. In contrast, exhaled metabolomic analysis indirectly reflects the activation state of trained immunity by detecting changes in specific metabolites (e.g., short-chain fatty acid derivatives) in exhaled breath. Circulating microRNAs, as molecular biomarkers in blood, show expression levels correlated with the degree of epigenetic modification in trained immune cells.^163^ For instance, upregulation of specific microRNAs may indicate enhanced training effects in alveolar macrophages.

The development of precise regulation tools is central to improving the safety and efficacy of trained immunity interventions. Traditional regulation methods often suffer from broad action ranges and insufficient specificity. Optogenetically controlled epigenetic editors can precisely regulate the expression of specific genes within immune cells through photodynamic control,^164^ enabling spatiotemporally precise activation of trained immunity. Nanocarriers targeting lung immune cells can deliver training adjuvants or metabolic modulators specifically to target cells like alveolar macrophages and dendritic cells,^165^ minimizing impacts on other tissues and reducing the risk of non-specific inflammation.

The application of organoid models in trained immunity mechanism research and drug screening provides convenience for both basic research and clinical translation. Traditional cell culture models struggle to simulate the complex *in vivo* environment, while animal models have species-specific limitations. Lung organoid–immune cell co-culture systems can recapitulate the structural characteristics and intercellular interactions of lung tissue. For example, they allow observation of dynamic interactions between alveolar macrophages and epithelial cells within the organoid system or evaluation of the regulatory effects of traditional Chinese medicine active components on trained immunity.^166^^,^^167^ Such models not only reveal the mechanisms of trained immunity more authentically, but can also significantly shorten drug screening cycles, aiding in the development of novel trained immunity modulators.

#### Clinical translation

In the clinical translation of trained immunity, clinical trials for trained immunity interventions targeting different respiratory diseases are gradually being initiated. BCG, a classic trained immunity inducer, is entering the exploratory stage in clinical trials investigating its role as an adjunctive therapy for lung cancer, aiming to enhance the killing activity of immune cells in the tumor microenvironment by activating innate immune memory. In the treatment of COPD, the application of metabolic modulators represents a significant breakthrough. These agents can modulate the metabolic state of alveolar macrophages,^168^ reversing disease-associated abnormalities in trained immunity, and alleviating airway inflammation and lung function decline. Related early-phase clinical trials have preliminarily verified their safety and potential efficacy.

Establishing respiratory disease risk stratification and personalized treatment plans based on trained immunity characteristics is a core direction for enhancing the precision of clinical interventions. The trained immunity status varies significantly among patients. Some asthma patients may exhibit features of over-activated trained immunity, while elderly COPD patients often experience a decline in trained immune function. This variation directly influences disease progression trajectories and treatment responses. Research indicates that peripheral blood immune cells from COVID-19 convalescents exhibit epigenetic signatures of trained immunity,^147^, ^169^ distinct from healthy individuals without the disease. This suggests that detecting trained immunity-related biomarkers (e.g., specific cytokine levels, epigenetic features of immune cells) in a patient’s peripheral blood could stratify patients into different risk categories, enabling the formulation of individualized plans to maximize therapeutic efficacy and minimize adverse reactions.

Exploring synergistic strategies combining trained immunity with existing treatment modalities provides a new optimization direction for respiratory disease therapy. Antibiotics are commonly used for treating respiratory bacterial infections, yet misuse can lead to antibiotic resistance,^170^ microbial dysbiosis,^171^ or secondary infections.^172^^,^^173^ Trained immunity, by enhancing macrophage phagocytic capacity and bactericidal activity, could potentially shorten antibiotic treatment duration and reduce the risk of resistance development. In the treatment of autoimmune respiratory diseases and their complications (e.g., upper respiratory infections), while immunosuppressants control inflammatory responses, they may also compromise host anti-infective defenses.^174^ In such scenarios, incorporating milder trained immunity interventions might help retain necessary innate immune defense functions while maintaining immunosuppressive efficacy, thereby reducing infectious complications. In-depth research on such synergistic strategies holds promise for expanding the application scenarios of trained immunity in future clinical practice.

#### Emerging biomarkers in respiratory diseases

The lack of clinically applicable biomarkers remains a major barrier for translating trained immunity into respiratory disease management. Recent advances, however, suggest that non-invasive and circulating biomarkers may provide feasible solutions.^175^ Exhaled breath metabolomics enables real-time assessment of airway metabolic activity and inflammatory status.^176^ Alterations in short-chain fatty acids, lactate derivatives, and lipid metabolites have been associated with trained or exhausted innate immune states in chronic lung diseases.^177^ In parallel, circulating microRNAs have emerged as stable biomarkers reflecting epigenetic and metabolic reprogramming in innate immune cells. Specific microRNA signatures correlate with monocyte activation, inflammatory memory, and disease severity, highlighting their potential utility for patient stratification and treatment monitoring.^178^ Integration of these biomarkers into clinical workflows may facilitate precision modulation of trained immunity and improve therapeutic outcomes in respiratory diseases.

## Conclusion

This review systematically delineates the central role of trained immunity in respiratory system diseases and its therapeutic prospects. In respiratory diseases, this mechanism exhibits a pronounced dual nature. Its protective function lies in its capacity to enhance the host’s broad-spectrum defense against subsequent infections and may also assist in anti-tumor immunity. However, dysregulation of this process can drive pathological progression. In response to this mechanism, diverse intervention strategies have been proposed, aiming to steer trained immunity toward beneficial outcomes. These strategies encompass vaccine induction, metabolic modulation, natural product application, and advanced cellular and genetic technologies, while a healthy lifestyle also plays a crucial role in maintaining equilibrium.

The field currently faces challenges such as achieving specific regulation, developing clinical assessment tools, and ensuring long-term safety. Future research must leverage novel technologies to deepen mechanistic understanding and drive the translation of theoretical concepts into clinical practice. Ultimately, this endeavor holds the promise of providing a new paradigm for the prevention and treatment of respiratory system diseases.

## Funding

This work was supported by the Noncommunicable Chronic Diseases-National Science and Technology Major Project (No. 2025ZD0549100), National Natural Science Foundation of China (No. 82241049, 82172285, 82572590 and 82550117), and the 1·3·5 Project of Excellent Development of West China Hospital of Sichuan University (No. ZYYC24002).

## CRediT authorship contribution statement

**Szu-yu Lee:** Writing – review & editing, Writing – original draft. **Jing Li:** Writing – review & editing, Writing – original draft. **Xikun Zhou:** Writing – review & editing, Writing – original draft, Conceptualization.

## Declaration of competing interest

The authors declare that they have no known competing financial interests or personal relationships that could have appeared to influence the work reported in this paper.
